# Crowdsourcing Team Formation With Worker-Centered Modeling

**DOI:** 10.3389/frai.2022.818562

**Published:** 2022-05-27

**Authors:** Federica Lucia Vinella, Jiayuan Hu, Ioanna Lykourentzou, Judith Masthoff

**Affiliations:** Human Centred-Computing, Department of Information and Computing Sciences, Utrecht University, Utrecht, Netherlands

**Keywords:** crowdsourcing, agent based modeling, social computing, self-organization, team formation

## Abstract

Modern crowdsourcing offers the potential to produce solutions for increasingly complex tasks requiring teamwork and collective labor. However, the vast scale of the crowd makes forming project teams an intractable problem to coordinate manually. To date, most crowdsourcing collaborative platforms rely on algorithms to automate team formation based on worker profiling data and task objectives. As a top-down strategy, algorithmic crowd team formation tends to alienate workers causing poor collaboration, interpersonal clashes, and dissatisfaction. In this paper, we investigate different ways that crowd teams can be formed through three team formation models namely bottom-up, top-down, and hybrid. By simulating an open collaboration scenario such as a hackathon, we observe that the bottom-up model forms the most competitive teams with the highest teamwork quality. Furthermore, we note that bottom-up approaches are particularly suitable for populations with high-risk appetites (most workers being lenient toward exploring new team configurations) and high degrees of homophily (most workers preferring to work with similar teammates). Our study highlights the importance of integrating worker agency in algorithm-mediated team formation systems, especially in collaborative/competitive settings, and bears practical implications for large-scale crowdsourcing platforms.

## 1. Introduction

Online, on-demand, and large-scale work, also called crowd work, is increasingly gaining traction. For more and more people, this new labor model is no longer used just for side “gigs” but as a primary source of income. Companies are also shifting toward elastic labor models, increasing their share of crowd workers in favor of a full-time workforce (LLP, 2020). The pandemic accelerated this trend, forcing many people to re-skill, up-skill, and to work with unfamiliar and distant collaborators, especially in the form of crowd work (Barnes et al., 2015; De Stefano, 2015; Manyika et al., 2016). Besides small, straightforward tasks, also known as micro-tasks (Difallah et al., 2015), such as image recognition, captcha annotation, and translation, crowds are now increasingly being involved in generating solutions to difficult or “wicked” problems, such as climate change mitigation, disease spread prevention, or rapid innovation generation. Tasks of this sort also called macro-tasks (Khan et al., 2019), tend to be complex and ill-structured, with multiple knowledge interdependencies and no straightforward solution. Because of their complex and open-ended character, these tasks typically require collaboration among workers of different skill sets and knowledge backgrounds. While micro-tasks lend themselves to being solved quickly and are therefore short-lived and affordable, macro-tasks frequently urge interdisciplinary collaboration, require more time, and are more challenging due to their breadth of scope.

Driven by the need to innovate and stay ahead of competition, companies increasingly make open calls for solving creative challenges through platforms such as OpenIdeo (Lakhani et al., 2012) and InnoCentive (Lakhani and Lonstein, 2008), where teams of crowd workers compete for prizes (Betts and Bloom, 2014). Another type of commercial task, which highly depends on the successful collaboration of crowd teams are online creative hackathons, for example those dedicated to video game development. Events such as the Global Game Jam gather thousands of online participants, including artists, developers, marketers, who form teams to compete for the best game product; sustainable game production in this case directly depends on the participants' ability to find the right group to work with Whitson et al. (2021). Aside from pure commercial interest, crowd team formation is also at the core of governmental initiatives. With the profound societal changes brought by the COVID-19 pandemic, grassroots entrepreneurship efforts have increased to stimulate economies and slow down infection rates. With 9,000 participants from 142 countries and 49 states, the Massachusetts Institute of Technology (MIT) COVID-19 Challenge is the most recent exemplary attempt addressing immediate needs with rapid innovation through a series of virtual hackathons involving *ad-hoc* teams of remote participants (Ramadi and Nguyen, 2021).

To coordinate the efforts of such workforce, crowdsourcing research has started to look into team formation algorithms as automated, scalable solutions. Routinely, team formation algorithms match workers according to objectives such as interpersonal compatibility (Lykourentzou et al., 2021) and social network connectivity (Liu et al., 2015; Rahman et al., 2019). One of the limits of computed team formation solutions—which we address in this study—is the omission of the workers' preferences and evolving relationships in the algorithmic objective function. In other words, workers have no say in whether they want to stay in a team chosen for them, and who they will work with. Team formation algorithms usually collect the workers' profile features *before* the task begins (Liu et al., 2015; Rahman et al., 2019), but then do not adjust to the workers' utilities and pay-offs *during* the collaboration. Although the workers' attributes are gathered only once, they are often assumed to suffice for the formulation of optimal teams. As a result, algorithms often fail to capture covert features such as temporal team dynamics information, collaboration preferences, intra-group compatibility, and individual risk appetites; features that play a key role in teamwork success (Degli Antoni et al., 2021). Aside from profiling information, team formation systems have recently started to factor social network properties in their objective functions, bringing together teams based on their network tie strength (Salehi and Bernstein, 2018) mutating as the collaboration evolves. However, in this case too, the system does not adapt its decisions based on worker feedback concerning the enforced rotations, and it does not account for cases where the workers' ties deteriorate or even break. In reality, however, individual team member agency makes up a significant portion of whether a team will be able to perform successfully or not, and removing it could mean reducing the adequacy and fairness of the team formation system.

Concerns about the poor representation of worker agency in automated team formation solutions are starting to surface. Recent research shows that purely top-down solutions result in rigid team structures and workflows that stifle creativity and initiative-taking, and inhibit workers from adapting their problem-solving strategy to the task needs, which, in turn, is detrimental for complex and open-ended tasks (Retelny et al., 2017). Forcing workers to work with specific people can also cause psychological fatigue and discomfort, reduce user autonomy, alienate workers, and lead to less-than-optimal collaboration (Rasmussen and Jeppesen, 2006; Lawler and Worley, 2009). A growing number of studies are starting to propose ways to incorporate worker agency, including preferences but also unconscious drivers, into crowd work settings, so as to directly and positively advance teamwork quality, efficiency, and well-being. Gaikwad et al. (2015, 2017) and Whiting et al. (2017) show that incorporating elements of open governance has been found to promote trust between workers and task providers. Yin et al. (2018) show that trusting workers with the work schedule increases the number of tasks completed without compromising quality, with workers actually willing to forego significant pay to control their working time. Specifically to the domain of collaborative work, Lykourentzou et al. (2016b) use a technique known as team dating, where people meet with candidate teammates in rapid succession before deciding to settle into teams. Although their solution integrates agency only indirectly, by forming teams based on peer evaluations of the intermediate team dates, this study shows that accounting for worker feedback during team formation can have a positive effect on team performance and satisfaction. Looking at research preceding the online crowdsourcing and open collaboration movements (Jackson, 1983; De Dreu and West, 2001), we also spot fundamental evidence on the importance of allowing workers' agency in teamwork such as through minority dissent and participation in decision making. Granted autonomy, individuals not only produce improved results (Gilson and Shalley, 2004; Costa et al., 2018), but also exhibit healthier mental states associated with self-governance, feelings of empowerment, reduced stress, sense of ownership over their work and ideas, and increased group interdependence and cohesion (Carless and De Paola, 2000; Rasmussen and Jeppesen, 2006; Haas and Mortensen, 2016). In this study we are interested in exploring how more worker-centered and bottom-up team formation compares to the prevalent approach of forming teams in a top-down and purely algorithm-driven manner. We do so by modeling and comparing three team formation systems, namely a (i) **fully bottom-up** system, where we model algorithm involvement to be minimal and team formation to lie almost exclusively on worker decisions, a (ii) **fully top-down** one, where we adapt a latest state-of-the-art team formation algorithm (Salehi and Bernstein, 2018), (iii) and (iv) a **hybrid** system, which borrows elements from the previous two. Although the three system models all aim to tackle team formation, their difference lies in the level of agency they permit and the degree of algorithmic mediation they enforce during team formation.

The fully bottom-up system (which we call SOT from Self-Organized Teams) is represented by two models. The first model, called **Radical SOT (R-SOT)**, prioritizes individual worker over team preferences of new teammates, and dismantles an existing team if at least one of its members decides to leave. The model focuses on facilitating novel interactions between the workers and leads to radical restructures of the collaboration network. The second model, called **Conservative SOT (C-SOT)**, facilitates bottom-up team formation in a less radical manner, since it prioritizes team over solo worker agency. In this model, teams looking for members have priority over individuals, and a team remains together as long as two of its members wish to keep collaborating. This model prioritizes majority consent over minority dissent. For the top-down model, we adopt **Hive**, a community-based team formation algorithm by Salehi and Bernstein (2018). Hive was chosen as it is a state-of-the-art algorithm and it represents the latest trend in top-down team formation approaches which adapt their decisions during the task rather than making them only once in the beginning. Briefly, Hive uses social network information to rotate people across teams so as to balance tie strength and network efficiency, and computes teamwork quality whilst rotating teams according to a stochastic search suited to minimize algorithm complexity. Finally, combining bottom-up and top-down approaches, we propose and add to the comparison a third hybrid system model named **HiveHybrid**. The model combines worker agency with algorithmic mediation. In this model, the algorithm offers to rotate workers according to the Hive system's objective function, but workers have then the option to accept or decline these proposals based on whether they are predisposed to break ties with their teammates or not (depending on their assessment of team reward and their personal risk appetite). In HiveHybrid, the workers' preferences play as much of a role in team formation as the coordinating algorithm. We run a comparative study, using agent-based simulations on the scenario of team formation for a creative game development hackathon, to evaluate differences in teamwork quality across these three team formation models. We focus on answering the following research questions:

1. **RQ1. How does bottom-up team formation compare with top-down and hybrid approaches?** We first compare the three team formation system models on the teamwork quality they yield, since quality is the primary and typical concern of crowdsourcing research, platforms, and clients. We use three metrics, namely the best, average, and worst teamwork quality, which are relevant depending on the requirements and constraints of the specific crowd work use case one is interested in.
2. **RQ2: How do population behavioral tendencies affect the outcome of bottom-up online teamwork?** Since bottom-up systems are more influenced by the participating workers' attributes, tendencies, preferences, and decisions than top-down ones, we systematically evaluate the effects of certain worker population's attributes on team performance. The objective of this evaluation is to help future crowdsourcing systems design incentives or countermeasures for different expected population behaviors, concerning team exploration tendencies, population size, and tendencies toward teamwork diversity. To systematically evaluate the effects of each of these attributes, we break down this research question into the following three sub-questions:
  - **RQ2.1: How do different risk appetites affect teamwork output in bottom-up models?** Workers with a high risk appetite tend to leave teams and rotate more often (preference for exploration) compared to workers with a lower risk appetite who tend to form more lasting teams (preference for exploitation). Risk appetite is expected to affect teamwork quality as it affects the number and structure of the self-organized teams. As a personal attribute, risk appetite is not only to influence the frequency of changes but also the preference of tasks (i.e., some workers might prefer tasks that are higher paid but less likely to be completed successfully), however, for simplicity, we have focused on one task type for this study.
  - **RQ2.2: How do different worker population sizes affect teamwork output in bottom-up models?** Evaluating the effects of changes in the population size helps to understand how changes in crowdsourcing collaborative participation affects the workers' search space, coordination costs, and teamwork quality.
  - **RQ2.3: How does homophily, i.e., the tendency to prefer working with similar teammates, affect teamwork output in bottom-up models?** Homophily is known to affect social interactions as people tend to choose (work with) partners based on shared physical and cultural cues (Haun and Over, 2015). Evaluating the effects of different homophily thresholds of the participating worker population on quality can facilitate the evaluation of whether certain explicit system incentives are needed to encourage workers to join forces with different collaborators or not.

Our results contribute to the development of future crowdsourcing tools for team formation that can be adapted—with the introduction of more or less degrees of agency—to the needs of the particular use case and the characteristics of the specific worker population involved. For one, we observe that **self-organization supports the formation of competitive teams**. In use case scenarios where innovation is key, a system capable of preserving worker agency can be a good return-on-investment for organizations that leverage competitive skills. Inspiring exploration across a large pool of curious workers seems to be an adequate strategy for forming competitive teams in bottom-up settings; so is the emancipation of team similarity where workers favor teammates of similar cultural and demographic attributes when workers have full control over team rotation. On the contrary, usage scenarios where it is more important to maintain fairness than performance, could benefit more from algorithmic-mediated team formation solutions to explicitly moderate the segregating tendencies we observe in fully bottom-up models. In this case, our results indicate that a hybrid system such as HybridHive constitutes an advantage over either fully top-down or fully bottom-up models, since it balances the global distribution of resources with worker agency mediating micro behavior through macro structures.

The rest of this paper is organized as follows. We first provide an overview of existing team formation approaches focusing on the collaborative crowdsourcing domain (Section 2). Afterward, we dive deeper into the modeling components that make the three team formation systems examined in this study (Section 3). Next, we present the results of the simulations comparing the three systems, mapped to the relevant research questions (Section 4). We then proceed by discussing the applicability and relevance of the findings (Section 5), followed by reasoning on the limitations of this study (Section 6). We conclude the paper with the main findings, key messages, and final remarks (Section 7).

## 2. Related Work

### 2.1. Team Formation Algorithms for Managing Online Work

Broadly speaking, the Team Formation Problem (TFP) is the problem of allocating a set of people to subsets, referred to as teams, according to a set of criteria that vary depending on the application area (Juárez et al., 2021). As illustrated in Juárez et al. (2021)'s recent review and taxonomy, TFP research has been persistently increasing over the past ten years. The problem encompasses a wide variety of applications, ranging from the assignment of students to study groups, to the distribution of patients to hospital rooms, and from the assignment of reviewers to papers, to the composition of teams for collaborative work purposes. In this paper, we focus on team formation for online work and, in particular, large-scale crowd participation in collaborative work. The research community has mostly focused on designing algorithms that ensure the quality of digital work by orchestrating people in a *top-down* manner, mainly with the objective to optimize costs. A recent extensive bibliometric analysis of 268 articles on crowd work task recommendation (Yin et al., 2020), covering the period of 2006–2019 (practically since the onset of crowd work) confirms the above, revealing that the largest and most durable research clusters focus on forming teams to optimize the task's budget, using methods such as dynamic programming, routing, and allocation. Similar methods are standard practice in operational research (Taha, 2013), an area traditionally geared toward optimizing supply chain management and manufacturing.

#### 2.1.1. Static Team Formation Models: Making Decisions Only Once

The problem of forming optimal teams is generally 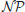-hard, and for this reason the majority of team formation algorithms make their decisions in a deterministic fashion and only once **at the beginning of the task**. The algorithm's intervention in these cases ends with one-off team formation decisions, after which the teams remain stationary, indisputable, and irreversible. Commonly used team formation systems typically bank on pre-existing workers profiling data, such as skills, availability, or hourly wage to estimate teamwork dimensions including expertise complementary (Rahman et al., 2019), team costs (Liu et al., 2015), and team roles (Retelny et al., 2014; Valentine et al., 2017). Subsequently, the algorithms feed this data to machine learning or combinatorial optimization models to produce (near-)optimal solutions. An example of such an approach is the work by Rahman et al. (2019) proposing an algorithm that relies on worker skills, wage, and pairwise affinity to match workers with teams and teams with tasks. Other examples include the work by Yu et al. (2019) using the Hungarian algorithm to calculate matches based on skill, task complexity, and active time, and the work by Ahmed et al. (2020) exploring crowdsourcing sequential arrival with the objective to maximize teams' utility and diversity.

Besides handling team formation as a combinatorial optimization problem, there are other ways that crowdsourcing team formation problems have been thought of. An example is the work by Liu et al. (2015) operating through a mechanism design approach that proposes a task pricing algorithm seeking to assemble crowd teams on the basis of costs and skills. This work looks at worker truthfulness in the bidding process as a desirable property of the model, where incentive compatibility results in the preferred dominant strategy. Models of this kind rely on pre-calculated assumptions and deterministic predictions to make their team formation decisions and are especially useful in settings where task requirements are well-defined and known a priori, and worker characteristics are immutable. For these tasks, the use of pre-calculated teams permits to scale-up and compute solutions that are both computationally efficient and high-quality (Avis, 1983). However, static models do not appraise changes in the collaborative environment, for example, changes in the workers' preferences and affinities as they work together, the evolution of team dynamics, or changes in the task requirements (e.g., expertise needed) over the course of the collaboration (Ananny, 2016; Faraj et al., 2018). Consequentially, they risk creating rigid team structures that cannot optimally address tasks of evolving complexity.

#### 2.1.2. Dynamic Team Formation Models: Adapting to Change

Recently, research has started looking into adaptive algorithms that make their team formation decisions **during the task**, as the collaboration unfolds. In this direction, Zhou et al. (2018) propose an algorithm using multi-armed bandits with temporal constraints, which explores the trade-offs among various dimensions of team structure, such as interaction patterns or hierarchies. By letting each bandit observe team performance and choose which arm to use next, the algorithm decides when and how to make changes in the structure of each team. In another example, Retelny et al. (2014) and Valentine et al. (2017) propose Foundry, a crowd management system that assembles workers into role-based teams. Although workers can request changes in the original teams, the final decision is made by a small number of experts and the task requester. Aside from skill sets, budget, and time, a small set of recent studies has started proposing team formation algorithms that harness social network qualities such as connectivity (Salehi and Bernstein, 2018), centrality (Hasteer et al., 2015), and marginality (Wang, 2020), as non-trivial parameters affecting teamwork performance across time. In this direction, Jiang et al. (2019) propose a team formation algorithm that instead of forming artificial teams, based on the individual teammates' skills, cost, or other features, utilizes groups that have been naturally organized through social networks, and allocated them to tasks in a priority-based manner based on their capacity to address the task. In the same line, Wu et al. (2021) propose a graph-based algorithm that estimates the accuracy of allocating a group of workers to a task, by joining the factorized matrixes of the workers' social network connections with their work history of on tasks.

Relevant to this study is the work of Salehi and Bernstein (2018). It envisages an online model (Hive algorithm) that balances two competing forces in team formation optimization: network efficiency and tie strength among the different worker pairs. It conceives crowdsourcing team formation as a graph partitioning problem where disjoint subsets (teams) benefit from strong ties but suffer from a lack of connectivity within the collaborative environment. This approach is an attempt to reconcile familiarity (obtained when relationships remain constant over time) and serendipity (spurred when breaking old ties and forming new ones). It handles team formation problems sequentially and in a stochastic fashion, juxtaposing top-down appointed team rotation with a series of collaborative stages of crowdsourcing work. It mediates team rotation by picking probabilistic moves at every round in keeping with a combination of tie strength and network efficiency. Rotating teams in crowd open collaboration resulted to be remarkably successful in connecting diverse perspectives. However, the same model provoked discomfort as workers could not determine by themselves the outcome of the match and could not depart from inefficient teams or decide to remain in the preferred one. We use Hive as a state-of-the-art representative benchmark of top-down work coordination in simulated scenarios. Although the above algorithms adapt to changes in team performance and task requirements that may occur over time, they are still fully top-down mechanisms that infer their decisions without actively engaging workers in the decision-making process.

In summary, relying on top-down coordination to form teams presents clear limitations. First, it limits the breadth of attainable work to tasks that the algorithm can decompose and assign to workers according to predefined criteria. For this reason, top-down crowdsourcing team formation solutions are ideal for tasks that are usually well-structured, with known interdependencies, and clear knowledge boundaries. However, for creative complex tasks and innovation generation they still tend to ignore worker self-organizing abilities and under-cater work flexibility. Subsequently, they fail at empowering crowd workers and drastically limit personal development opportunities (Roy et al., 2013; Schriner and Oerther, 2014). Ergo, another major limitation of top-down solutions—especially in crowdsourcing collaborative spaces—is the workers' confinement and isolation within the collaborative environment where algorithms direct and workers execute (Berg, 2015; Smith and Leberstein, 2015; Popescu et al., 2018; Gray and Suri, 2019). Furthermore, the pay-per-work model leads to the commodification of online work and online workers (Wood et al., 2019). It also means that workers must bear “work-for-labor” costs, i.e., costs for activities like breaks, training, or waiting for work—which are necessary to perform the task—but they are not part of the work itself (Berg, 2015; Florisson and Mandl, 2018) as they are still treated as separate entities from the collaboration and the end-result. For these reasons, ethical issues also arise (Silberman et al., 2018) concerning the labor conditions of crowd workers, their rights and legal status (Deitz, 2016), and “lock-in” phenomena where workers are tied to platform monopolies and non-transferrable profile information (e.g., performance history). In the last years, more and more researchers are raising critical voices (Smith and Leberstein, 2015; Gray and Suri, 2019) regarding the need to shift away from the canonical top-down crowdsourcing team formation systems and give workers agency, control, and self-determination capacity.

### 2.2. Self-Organization in Team Formation: Mediating Through Guidance

The term self-organization is present across several managerial and scientific fields spanning from software development communities to complex systems and natural science. The term describes the emergence of spontaneous processes and interactions between entities of originally disordered systems (Yates, 2012; Anzola et al., 2017). In team formation, self-organization usually describes the behavior of individuals as they form groups and collaborate autonomously and without pre-defined leadership. In software development, the term self-organization typically indicates the distribution of workload among teammates who flexibly shift responsibilities and partake in decision-making (Highsmith, 2009). Self-organized teams are known to benefit from transferable authority (Moe and Dingsøyr, 2008), as well as from robust and adaptable collaborative networks (Marzo Serugendo et al., 2003). The work of Lykourentzou et al. (2016b) explores the self-organization phenomena in the crowdsourcing domain in the way it affects teamwork. In their study, unfamiliar workers try out potential teammates before settling into teams, thus self-organizing into reciprocal work groups. Their results show that handing decision-making power to crowd workers increases performance compared to top-down team allocation. Further, as shown in Rokicki et al. (2015), when applying self-organization to crowd teams reward systems, ergo when allowing people to decide upon reward distribution, the self-governing approach results in fairer compensation than conventional top-down reward systems.

However, simply relying on self-organization as an emerging, non-controlled property is not enough for digital labor systems. For one, the need to adhere to financial and quality targets can suffer from purely self-organized means. Entirely autonomous teams can risk overspending on resources and coordination time, two essential aspects of teamwork. Consequently, we evaluate the efficacy of **guided self-organization** as a resolution between central control and self-governance. This relatively new approach (Prokopenko, 2009) aims to regulate self-organization in dynamic complex systems by combining task-independent global goals (e.g., autonomy, fairness, governance) with task-dependent constraints (e.g., costs, efficiency) on local interactions. Up to now, this approach has been thoroughly researched in robotics (Martius and Herrmann, 2012; Nurzaman et al., 2014). As for crowd work, guided self-organization is the golden mean between safeguarding worker autonomy and protecting digital work platforms from disintermediation (Jarrahi et al., 2020). In the past, the principles of guided self-organization (albeit under a different name) have touched upon collaborative knowledge production (Lykourentzou et al., 2010) and crowdsourcing teams (Lykourentzou et al., 2019). These studies indicate that guided self-organization is a potentially effective coordination model for crowd collaboration in a manner that is distributed, efficient, and fair. In our study, guided self-organization is represented by a hybrid model which combines bottom-up self-organization with top-down community-based team formation.

## 3. Methodology

**Figure 1:**
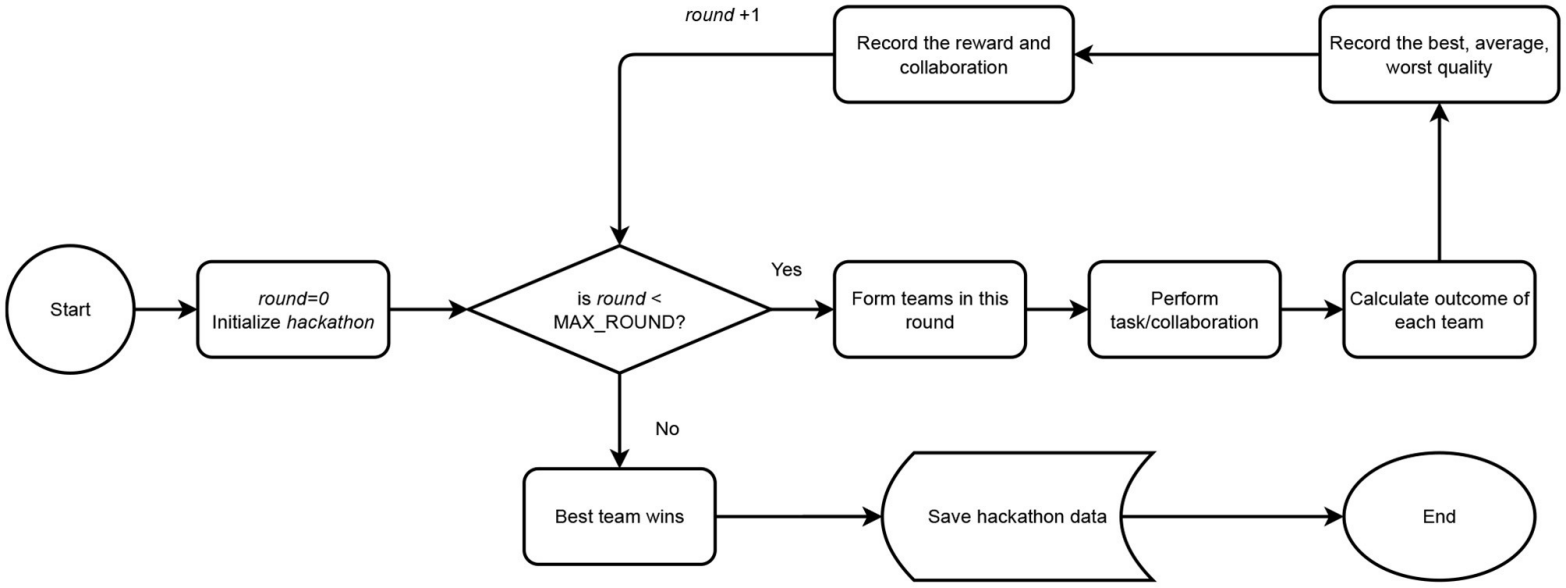
System architecture displaying the steps taken by the system in accordance with the hackathon design starting from the initialization of the agents and proceeding to the formation of teams assessed across ten rounds.

In this study, we attempt to re-create and predict emerging properties of online crowdsourcing collaborative settings where the actions of multiple workers—and the intervention of team formation approaches—affect teamwork and team output. Our simulation consists of three components: the **setting** (Section 3.1), the **agents** (Section 3.2), and the modeling of the **work coordination models** (Section 3.3). These are fundamental parts of the simulated scenario and exhibit behavioral properties, functional objectives, and constraints typically present in real-world crowd collaborative systems. Figure 1 showcases the hackhathon system architecture.

### 3.1. Setting

Our simulation setting is a cycle-based online crowdsourcing hackathon. Online hackathons represent collaborative scenarios where several remote crowd workers of different backgrounds can gather in teams to create projects and compete for prizes. Even though hackathons have originated from the software development community (e.g., cybersecurity, game jams, open-source development, and operating systems) (Nolte et al., 2018), they are increasingly popular in other domains such as crowdsourcing innovation (Temiz, 2021; Wang et al., 2021). Further, as society faces progressively more global challenges, the help of citizens—and more broadly crowds—is also being used to find solutions to universal problems such as carbon emissions, household waste, and deforestation through collective idea generation (Monsef et al., 2021).

In our scenario, the (hypothetical) company recruits participants (game developers, marketers, designers, testers) online from popular crowdsourcing platforms (Amazon Mechanical Turk, Upwork, etc.) or other venues (e.g., creative hubs)^1^ and retains them until the end of the event (Section 3.2). During the first round, workers are initially grouped randomly into teams of four and then they are required to collaborate for a number of consecutive rounds, which for our scenario is set to ten (Section 3.4). Depending on the approach involved in the team formation process (Section 3.3) and the level of the workers' agency modeled in the system, workers may move to other teams voluntarily or by top-down means. At the end of each round, each worker is given a reward (which can be thought of in monetary terms, e.g., in US Dollars), based on the ranking of their team's quality compared with other teams using the reward function (Equation 1).


(1) $$\begin{array}{r} reward=\frac{n-j}{n-1},\text{for team of ranking}j, \end{array}$$

where *n* is the number of teams.

The product of each team is evaluated, using a quality function (described by Equation (2) and introduced in detail in Section 3.2.2, which simulates external evaluation by means of an external jury). At the end of the final (tenth in our simulations) round, the system automatically identifies the final best, average, and worst projects computed by means of the teamwork quality function (Equation 2).

### 3.2. Agents

Here we describe the modeling of the two key strategic agents of the team formation problem, namely the: (i) workers and their individual characteristics and (ii) the teams, consisting of multiple workers.

#### 3.2.1. Worker

For the simulation and in line with our working scenario, we focus on crowd worker profiles that can be involved in video game development in the context of a hackathon. We model worker attributes (Table 1) into two categories: (i) **manifest** (Section 3.2.1.1) and (ii) **latent** (Section 3.2.1.2) properties. Manifest attributes are those worker characteristics that are straightforwardly noticeable by others and can be captured into the profiling information of online team formation systems (Lykourentzou et al., 2021). These attributes are the workers' *knowledge domain, nationality, educational level*, and *age*. The latent attributes withhold worker characteristics that are not directly evident to others but that do affect the workers' compatibility, exploratory behavior, and competency. These latent characteristics are *personality, risk appetite*, and *expertise*. We distribute both manifest and latent attributes in relation to a set of probability functions based on previous work and modeled on the likelihood of occurring within a crowd population.

**Table 1:** Worker attributes observing their mutability, visibility, type, possible value, and distribution.

**Attribute**	**Possible attribute**	**Instantiation**	**Mutability**	**Visibility**
Knowledge domain	Developer; Designer; Marketer; Tester	Random uniform	Immutable	Manifest
Nationality	USA; India; Other			
Educational level	High school; Bachelor; Master or above			
Age (years)	<20; 21–30; 31–40; 41–50; 51–60; >60	<20 = 2%, [21..30] = 40%, [31..40] = 36%, [41..50] = 7%, [52..60] = 9%, >60 = 4%		
Personality	Dominant; Inspiring; Supportive; Cautious	D = 50%; I = 10%; S = 20%; C = 20%		Latent
Risk appetite	[0,1]	Beta distribution (β = 2, α = 2)		
Expertise	[0,1]	Beta distribution (β = 2, α = 2)		

##### 3.2.1.1. Manifest Attributes

1. **Knowledge domain**. This attribute captures worker expertise and is intended for the division of labor within a team. Following our working scenario on game development, we model four knowledge domains, namely: (1) Developer (typically a computer science specialist who creates software and application), (2) Designer (a game designer invested in software design, computer graphics, and animation), (3) Marketer (specialist in charge of monitoring market trends and creating advertising campaigns), and (4) Tester (worker in charge of playing the game to find errors and issues and evaluate the user experience). These domains are abstract representations of real-world work division in project-based teams and are relevant to scenarios where interdisciplinarity is vital to teamwork (Haeussler and Sauermann, 2020). All four knowledge domains manifest in the population with a random uniform distribution such that each trait has an equal probability of being expressed in the worker pool.
2. **Nationality**. This attribute imitates cultural differences in communication style, norms, and customs (Ortu et al., 2017) and may affect the workers' likelihood of seeking others similar to them (Centola et al., 2007). We model three nationalities as the most common among crowdsourcing workers (Difallah et al., 2018), namely: (1) USA, (2) Indian, and (3) Other nationalities. Just like the knowledge domain, nationalities are distributed randomly and uniformly across the population.
3. **Educational levels**. We model the workers' highest obtained educational qualification as: (1) High school, (2) Bachelor, or (3) Master or higher. We include the educational level in the working model for two main reasons. The first is that educational background is often a pivotal factor in hiring processes, including screening in crowdsourcing platforms such as AMT and Prolific (Prolific Team, 2021). The second reason is that, like social status, educational levels affect workers' preferences for teammates (McPherson et al., 2001) of similar or higher education. This attribute is also randomly and uniformly distributed in the worker pool.
4. **Age**. We model age in intervals [ <20; 21–30; 31–40; 41–50; 51–60; >60] to classify differences in work culture, viewpoints, and collective identity. Age may also affect worker choice of teammates, with workers tending to favor collaborators of similar age with whom they are likely to share similar attitudes and beliefs (McPherson et al., 2001). The age attribute is distributed in accordance with crowdsourcing demographic statistics by Difallah et al. (2018) where <2% are younger than 20 years old, ~40% are between 21 and 30, ~36% are between 31 and 40, over 7% is between 41 and 50, a little over 9% is between 51 and 60, while the remaining 4% is older than 61.
5. **Past average reward**. We model past average reward as the average of the rewards received by the worker through their previous team collaborations (as a reminder a worker's past reward per round is calculated using Equation 1).

##### 3.2.1.2. Latent Attributes

1. **Personality**. Using the DISC personality model by Marston (2013), we classify workers' approaches to leadership roles and team problems as being: (1) Dominant (D), (2) Inspiring (I), (3) Supportive (S), or (4) Cautious (C). DISC was selected as it is widely used specifically in work-related settings, for example during hiring processes (Furlow, 2000). Each trait influences a worker's attitude to teamwork and mimics interpersonal factors affecting team processes. Based on the study by Lykourentzou et al. (2016a), we factor workers' personalities in the teamwork quality calculation and bonus teams of equally balanced personality traits (Equation 2). The aforementioned study also provides us with the distribution of personalities in a typical crowd work population, as follows: 50% of the workers are of personality type D, 20% are of type S, another 20% are of type C, and the remaining 10% are of type I.
2. **Risk appetite**. This represents to what extent workers are willing to explore new teams. The concept takes from the exploration-exploitation trade-off dilemma (Berger-Tal et al., 2014) concerning the problem of choosing between conserving a state or exploring new ones. In this case, a worker's risk appetite is mutable and determines one's tendency to seek collaborators outside their teams. We model each worker's risk appetite as value in the [0, 1] range and distribute it across the population using a beta distribution probability function (Eugene et al., 2002). The beta distribution was chosen because it is bounded and can be easily modeled to illustrate various probability density functions (e.g., most workers having a low risk level with a long tail of high risk-workers, or vice versa).
3. **Expertise**. This attribute concerns the workers' level of ability in the knowledge domain in which they belong (Developer, Designer, Marketer, or Tester). It is not to be confused with education which is the formal training and schooling of the worker, which is used for computing the decisions taken by the workers on the basis of similarity. Expertise is modeled as a manifest attribute in the sense that, just like in real conditions, other workers (and the profiling system) can easily see *which* knowledge domain each other worker belongs to, but not *how good* the worker is in the specific domain. In our simulation, workers' expertise is treated as an immutable parameter and is distributed in the population with a beta probability distribution function (PDF) similar to a bell curve (with parameters α = 2 and β = 2), i.e., most workers are of average expertise in their respective knowledge domains, and less workers are either complete novices or complete experts.
4. **Homophily**. This attribute describes the degree to which workers tend to prefer working with people that are more or less similar to themselves. We model homophily as it is one of the most studied motivators for forming social ties (McPherson et al., 2001). This principle structures human connections and knowledge exchange as well as restricting social worlds and interactions through subjective preferences for similar nationality, age, education, etc. (McPherson et al., 2001). We model worker's homphily as a cosine similarity score between two workers' vectors consisting of the attributes knowledge domain, nationality, educational level, and age.

#### 3.2.2. Team

A team is a group of workers collaborating together for the duration of one or more rounds. Each team is a combination of the participating workers' attributes and their interactions, which affect the team output. Specifically, we model the output of each team, hereby referred to as **teamwork quality**, as a weighted sum of three elements, namely the team's: (1) skill, (2) interpersonal compatibility, and (3) size:


(2) $$\begin{array}{l} TeamworkQuality=\pi \times Teamskill+\mu \\ \;\times Interpersonalcompatibility\\ \;+(1-\pi -\mu )\times Teamsize, \end{array}$$

where:

1. **Team skill** is modeled as a weighted sum of the team members' expertise across the knowledge domains of the task, adjusted by a diminishing factor for repetitive expertise. Higher individual levels of expertise and higher coverage of the task's knowledge domains lead to higher team skill. We detail the modeling of the team skill element in Section 3.2.2.1.
2. **Interpersonal compatibility** is the degree to which the different teammates can work together harmoniously according to their work personality attribute. Higher coverage of the four personality types foreseen by the DISC test (D, I, S, and C) leads to higher teamwork quality. The presence of two or more members with personality type D (Dominant) lowers teamwork quality as it is known to produce clashes in collaborative crowd work settings (Lykourentzou et al., 2016a). We detail the modeling of this element in Section 3.2.2.2.
3. **Team size**. Team size affects teamwork quality, with teams above or below a certain threshold producing less-than-optimal results.

All three elements are measured in the [0, 1] range, which also bounds teamwork quality in the same range. The coefficients π and μ can vary depending on the desired modeling. For our specific simulation, we set them to π = 0.4 and μ = 0.4 (see Section 3.4).

##### 3.2.2.1. Team Skill

Team skill is calculated as the combination of: (1) *coverage of the task's knowledge domains* by the members of the team, and (2) their *expertise* levels per domain. We assume that workers' expertise contributes positively to teamwork and that the workers' skill diversity promotes team interdisciplinarity. In case there are several teammates with the same knowledge domain in a team, we apply a diminishing factor to their skill utility in descending skill order. For example, in a team where three workers share the same domain, the second most expert in that domain has their skill utility discounted by a diminishing factor (which for our simulations is set to 0.10). All other lesser experienced workers of the same domain have their skill utility diminished by the same factor squared. We also discount 10% to all first-met teammates to account for the fact that the process of getting to know others and adjusting to new ways of working together taxes teamwork. Team skill is therefore calculated as follows:


(3) $$Teamskill=\frac{1}{s_{t}}\times \sum_{d=1}^{n}\left(\sum_{i=1}^{c_{d}}expertise_{d,i}\times \theta^{i-1}\right),$$

where *s*_*t*_ is the size of the team, *n* is the number of total domains (four in this study), *c*_*d*_ is the number of workers in domain *d*, θ = 0.1 is the diminishing factor for multiple expertise, and *expertise*_*d, i*_ is the expertise of worker *i* in domain *d*.

##### 3.2.2.2. Team Compatibility

We recognize the diversity of personality types as a representative measure of team interpersonal compatibility. More specifically, according to the DISC personality model, the more diverse and balanced a team is in regards to their DISC personalities, the more performant that team will be. To this end, the best team in our modeling is one the members of which cover all four DICS personality types. Such a team is optimal because it avoids both work disputes (which take place in the event of too many dominant types) and a lack of cohesion (which happens in case of missing personality types; resulting e.g., in lack of leadership and work direction). We apply a penalty of factor 0.2 to teams that do not have the full DISC personality spectrum and a penalty of factor 0.4 to teams that have more than one worker with of Dominant personality type (D type). We bound team compatibility to a range [0, 1]. Finally, the team compatibility function looks as follows:


(4) $$TeamCompatibility=\;\{\begin{array}{cc} 0.4+0.2\times (n_{per}-1), & p_{D}<2\\ \;0.2\times (n_{per}-1), & p_{D}\geq 2, \end{array}$$

where, *n*_*per*_ is the number of all unique personality types, and *p*_*D*_ is the number of workers with a **Dominant** personality type within the team.

##### 3.2.2.3. Team Size

The team size is the third factor that affects team quality in our setting. Literature in small groups research (Moreland, 2010) tends to consider that groups of less than three people do not constitute a team, and that the minimum team size is three. The reason, is that dyads are more ephemeral than larger groups, and certain phenomena like majority/minority relations, coalition formation, and group socialization can only be observed in larger groups. At the same time, social theories underscore the importance of also having an upper critical mass for team collaboration, beyond which the collaboration effectiveness diminishes due to coordination costs (Marwell et al., 1988; Kenna and Berche, 2012). In our setting we apply a penalty factor of 0.1 to teamwork size utility for each additional worker above a maximum threshold of team size five and to each worker needed to reach a minimum team size of three. The team size penalty factor is expected to implicitly guide workers in the self-organized and hybrid approaches to form teams that are within an ideal size range between three and five and discourage them to settle for smaller or larger configurations. The team size function is calculated as follows.


(5) $$Teamsizeutility=\left\{ \begin{array}{l} \max(0,1-0.1\times (S_{MIN}-s_{team})),\\ \;s_{team}<S_{MIN}\\ \max(0,1-0.1\times (s_{team}-S_{MAX})),\\ \;s_{team}>S_{MAX}\\ \;1,\text{ otherwise}, \end{array}\right.$$

where *s*_team_ is the size of this team, and *S*_MIN_ = 3 and *S*_MAX_ = 5 is the minimal and maximal non-penalized size of a team, respectively.

### 3.3. Work Coordination Models

We distinguish and compare three work coordination models.

1. The first is a **top-down** model, where the state-of-the-art team formation algorithm Hive appoints teammates without any input from the workers. This strategy approaches TFPs in a controlled, directed, and centralized way. For this model we use the Hive algorithm (Salehi and Bernstein, 2018) designed to optimize team formation from a community-based, top-down approach.
2. The second is a **self-organized** model, where workers govern the team formation processes (grouping and dismantling), with certain rules concerning whether teams should dismantle in the event of minority dissent or not. This approach is inspired by the SOT framework (Lykourentzou et al., 2021) honoring workers' preferences of teammates through a voting system combined with a graph cutting algorithm. We foresee two SOT models called **Radical SOT (R-SOT)** and **Conservative SOT (C-SOT)**. While these two systems share the same bottom-up team formation principles, they differ in the way they handle team cohesion after changes in workers' preferences. Where R-SOT dismantles teams and constructs new ones each time a team member leaves (hence it radically changes team structures), C-SOT preserves teams by retaining their structure and allowing members to leave and join (thus conserving team states where possible). We chose to model two kinds of bottom-up strategies given that certain tasks favor one model over the other (for example radical vs. incremental innovation).
3. The third is a **hybrid** model; this is a mix of top-down and bottom-up team formation strategies where algorithmic intervention supports and is driven by worker feedback. In the hybrid model, network efficiency, tie strength, and workers' agency are combined into a unified system where teams are regularly dismantled in the event that at least one teammate wishes to leave.

#### 3.3.1. Top-Down Model: Hive

For the implementation of the top-down team formation strategy, we adopt Hive (Salehi and Bernstein, 2018), a crowdsourcing collaborative hierarchical team formation model for which community structures dictate network changes. Hive models workers as part of a collaboration graph, with workers as the nodes and the edges corresponding to prior worker collaborations. The objective of the algorithm is to regularly shuffle teams so as to bring together workers with different viewpoints (i.e., far away in the graph), while conserving tie strength. To do so, Hive groups people in teams with one fixed leader, and then intermixes the teams by rotating the people who are not leader. The original Hive paper does not specify how each leader is appointed, or which is the optimal team size to be used. To be able to apply Hive on our setting, we needed to make a decision concerning these two parameters; in both cases we made the decision that is the most favorable for Hive. Concerning team size, we used teams of five. This is the minimum team size for a worker team to have chances to cover all DISC personalities, plus one for the fixed team leader. This way, the Hive teams always have a leader and always have a chance to cover all DISC personality types, i.e., they have a chance to be optimal. Concerning leadership, we appoint the fixed (non-movable) leader of each team to be the team member who has a D personality type, if one such member exists. This way the Hive teams avoid being leaderless, which would result in less-than-optimal results.

In the event of too many workers of personality type D within the worker population, we randomly draw a subset of D-leaders equal to the number of teams. After all team leaders are assigned to their teams, we randomly match workers to the teams, in the same way that Hive randomly initializes the movable team members in the beginning of the task. With these modeling decisions in place, we proceed to model the Hive approach for our simulation. We first introduce the concepts and calculations of network efficiency and tie strength, which are central to the Hive algorithm. We then implement these metrics as part of Hive's objective function, and finally we describe the modeling of the stochastic search algorithm used to find possible team formation moves.

1. **Network efficiency**: The efficiency of a network describes how effectively it transports information across its nodes (Latora and Marchiori, 2001). Network efficiency is usually calculated as the average of the inverse of the minimal path length between every two nodes. By applying network efficiency to the simulation, we attribute the value 1 for all familiar ties (meaning ties linking workers who have collaborated in the past) and the value +∞ to those ties that do not share direct collaborative history. Formally, the network efficiency *NE* in the system is calculated as follows:

(6) $$NE(G)=\frac{1}{N(N-1)}\sum_{i\neq j\in G}\frac{1}{d_{ij}},$$

where *N* is the number of workers in the system and *d*_*ij*_ is the minimal path length between node (worker) *i* and node (worker) *j*.

**2. Tie strength**: Tie strength represents the level of closeness or affinity between two nodes of a network. In the simulation, tie strength is intended as the calculation of relationships between workers, and ties between nodes represent the workers' collaboration history. Following the Hive computation of tie strength, we apply a logistic function and dampening factor to represent incremental familiarly and progressive detachment, respectively.
  **(a) The logistic function** takes two parameters *k* = 8 and *x*_0_ = 0.2 used to simulate the rapid strengthening of relationships at the start of new collaborations (where tie strength is lower) and their slow increment over time.
  **(b) The dampening factor** captures the weakening of tie strength when workers no longer collaborate and are therefore not directly exposed to one another. For its calculation, we adopt the same value as Salehi and Bernstein (2018) (λ = 0.8).
3. **Objective function**: The objective function of Hive consists of combining network efficiency and tie strength; this is since, in the event of workers changing teams, network efficiency grows as new collaborations emerge (and information gets transported across the network) while tie strength decreases as there are less close relationships. We factor these parameters in the simulated model with a constant value α = 0.5 as described in Equation (7). Here, we normalize tie strength by a constant value *c* = 0.005.

(7) f(G)=α×TieStrength(G)+(1−α)×NetworkEfficiency(G)

4. **Stochastic search**: As also discussed by the makers of Hive (Salehi and Bernstein, 2018), effectively rotating teams in order to reach optimality is an extremely complex and non-uni-modular task [*O*(2^*N*^)]. We implement the stochastic search algorithm of Hive as described in the stochastic phase 1 (Algorithm 1) and phase 2 (Algorithm 2). In essence, the stochastic search algorithm finds a random valid move, i.e., it identifies which worker should move to which team, which carries greater utility than the previous move considered by the algorithm. It returns a solution when the search space has been exhausted or if the ϵ value is reached indicating the probability of stopping the search.

**Algorithm 1:**
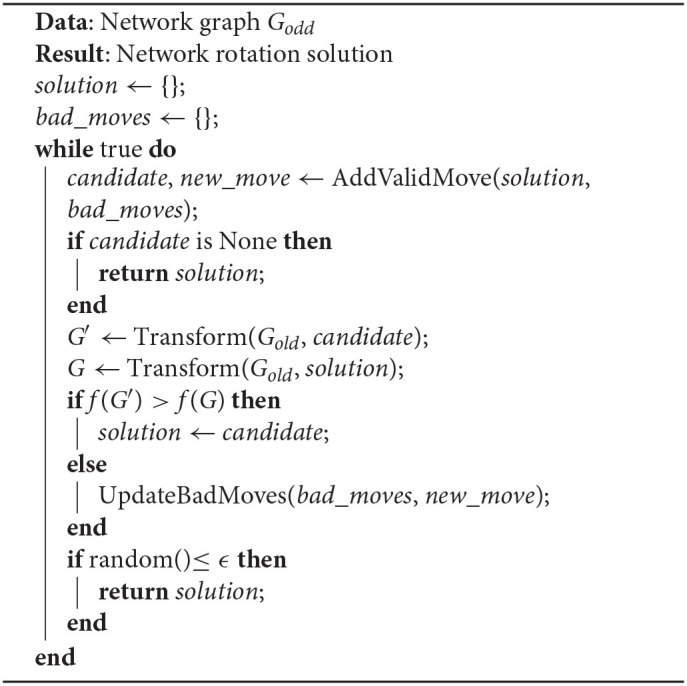
**Stochastic search algorithm**. The algorithm attempts to add as many valid rotations as allowed to the network graph, as long as the new rotation surpasses the current state of the objective function (7) and until either all moves are exhausted or a local maximum is reached (Salehi and Bernstein, 2018).

**Algorithm 2:**
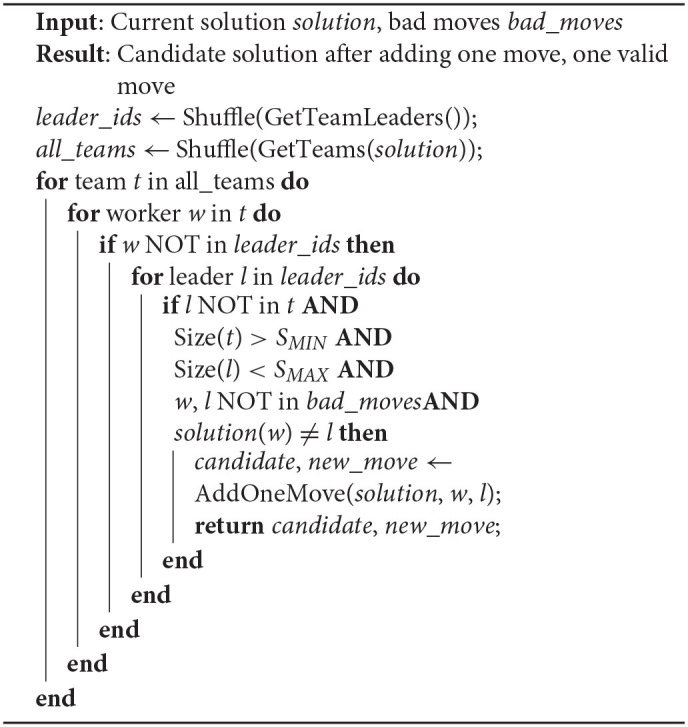
**Add valid move algorithm**. This algorithm loops for every team and for every worker (that are not team leaders), until it finds a worker and a team (represented by its leader) to meet the following five conditions: 1. the worker is not in the team; 2. current team size is within the system constraints; 3. target team size is within system constraints; 4. this combination is not a **bad move**; 5. this combination is new.

#### 3.3.2. Bottom-Up Model: SOT

In the bottom-up model, we simulate team formation on the basis of workers' preferences and affinities. In this context, teams strictly depend on what workers prefer and how likely they are to form effective teams with regards to their personality, knowledge domain, and team size. The simulation represents an abstraction of workers' behavior, performance, and constraints while they form teams in a self-organized manner. During each round workers are allowed to change teams after a deciding and searching phase.

1. **Deciding phase:** In this phase, workers evaluate the strength of their risk appetite against the reward they received in the previous round. The factor with the highest score (being it either risk appetite or reward Equation 1) determines whether that worker will decide to remain in the same team in the following round or whether they will join another team. A higher risk appetite stirs workers to leave and seek new coalitions in search for higher future rewards, whilst a lower risk appetite means that the worker will stay with their existing team even for lower rewards.
2. **Searching phase** Workers who decide to change teams proceed with the search phase, where they perform an evaluation of compatibility of the teammates and teams available to them. Specifically, during this phase, workers assess all possible combinations of teams of four by evaluating three other available workers based on a cosine similarity score of the four manifest attributes, i.e., the attributes of their co-workers that they can readily see (knowledge domain, nationality, educational level, and age). The cosine similarity score does not factor in the average past reward of the workers as it only deals with their manifest profiling attributes. However, in the event that two workers have the same similarity score, their average past reward is considered as a tie breaker. The search phase is further differentiated between the two bottom-up model variations as described below.
  (a) **Conservative SOT (C-SOT)** According to the conservative strategy, existing teams are given priority in choosing whether to admit new members or not. The C-SOT strategy considers existing teams as those that have worked together in the previous round and have at least two team members who decided to continue working together, during the deciding phase. For the rest of the workers and teams that do not fit into this description, the strategy considers these workers as available and unassigned entities. Then, the decision-making process is based on the homophily score (how similar the candidate team members are) constrained by a threshold (Section 3.4) determining the minimum similarity required to form matches. Teams recruit (are matched to) workers who have a similarity score higher than the threshold and higher than the rest of the available workers. If the similarity between existing teams and available solo workers is below the threshold (thus candidate teammates do not classify as sufficiently similar to any given team), the C-SOT model ignores the previously formed teams and matches available teammates based on the highest homophily score. The strategy then puts similar and available teammates into teams of four. In the case of equal similarity between workers or between workers and teams, the strategy prioritizes matches of the highest teamwork quality. Finally, in case that workers still cannot be matched, the C-SOT strategy puts those workers on hold until the next searching phase.
  (b) **Radical SOT (R-SOT)** While the C-SOT strategy attempts to preserve the existing teams' structures even though one or more members decide to leave, in the R-SOT strategy, a team is considered dismantled and all of its members are made available even if one worker from that team decides to leave. This means that available workers have higher chances of forming new teams since they are given access to more options. Besides this difference in the way of handling team deconstruction, the R-SOT follows the same approach as the C-SOT. It too assesses all possible combinations of similarities between four available workers and forms teams of the highest similarity score. In the event that no three workers are considered sufficiently similar to be matched, the R-SOT strategy strives to match workers with existing teams (intended as those that did not lose teammates in the deciding phase). If workers can still not be matched neither with a newly formed nor with an existing team, the team formation model leaves these workers on hold until the next searching phase.

#### 3.3.3. Top-Down and Bottom-Up Models Combined: HiveHybrid

Although bottom-up approaches to crowd TFPs—such as the SOT model—have certain advantages over top-down algorithmic solutions, their spontaneous nature and weak controllability can result in suboptimal solutions. Workers often cannot access the full array of options at once, mostly due to external constraints such as budget, availability, and time. More so, a system that fully relies on the workers' choices to form teams is susceptible to errors of judgment as workers evaluate others subjectively and cannot possess the same global overview of a centralized system. This means that workers cannot always judge the optimality of a match on the basis of both local and global objectives as their angle of vision is often restricted by what they can experience. This locality issue is even more present when the pool of workers is considerably large and workers are limited by how many people they can meet. Under the light of these inherent limitations of fully bottom-up solutions to crowdsourcing TFPs, we also model a blended approach inspired by Prokopenko (2009) who point that self-organization can (and should) be guided by algorithmic top-down mediation. Similar works (Lykourentzou et al., 2010, 2019; Martius and Herrmann, 2012; Nurzaman et al., 2014; Jarrahi et al., 2020)—either through conceptualization or real-life implementations—have proposed **guided self-organization** as the ideal strategy linking worker agency with algorithmic optimization. Our implementation of guided self-organization differs in the way it is applied to a simulated collaborative crowdsourcing scenario where workers are recommended by the algorithm whether to change teams or not. The HiveHybrid model is designed precisely to combine global objectives with local constraints in large-scale collaborative crowdsourcing. The system combines a bottom-up worker-centric SOT model with a top-down community-based Hive model. In the HiveHybrid, workers are allowed to decide to leave a team or remain as their choice is honored and optimized through a community-based team rotating algorithm. The algorithm identifies possible moves (rotations that would benefit the global objective function) and the workers can either accept or reject this offer if their appetite for exploration (risk appetite) indicates so.

### 3.4. Experimental Parameterization

Our simulation is designed to run a series of experiments where different populations and team formation models are tested and evaluated for their best, worst, and average teamwork quality. The following are the experimental parameters and corresponding settings used for this study. For the implementation of the Hive algorithm both as a baseline for top-down allocation and as part of the HiveHybrid model, we use the same parameters stated in the work by Salehi and Bernstein (2018).

1. **Experiment rounds (n)**: By rounds we intend the collaboration cycles during which workers form teams and collaborate. For this study, we used a fixed experiment of 10 rounds.
2. **Teamwork quality**:We calculate teamwork quality as follows. We first generate a batch of user agents as described in Section 3.2. For this batch, we run the simulation six times, each time extracting the best, average, and worst teamwork values, and then calculating the mean of those values to get the best, average, and worst teamwork quality of the batch. We repeat the process for thirty independent batch runs and average out the results. The procedure is designed to smooth out random fluctuations and yield less noisy simulation results.
3. **Population (x)**: The default population size is set to 20 workers. We consider this to be a rounded estimation of a basic size of participation required for creative tasks of this kind (online hackathon, expert crowdsourcing collaboration, etc.). Then, to examine generalizability, we gradually increase this number and experiment with larger populations ([30, 40, 50, …, 100]).
4. **Team size threshold (*S*_*MIN*_, *S*_*MAX*_)**: We constrain teams within a range of three (minimal size) and five (maximal size) teammates. We apply these threshold since we expect smaller teams to be hindered by a shortage of knowledge domains and personalities while larger teams to be taxed by coordination and communication costs, as explained in Section 3.2.2.3.
5. **Risk appetite (β)**: We represent worker's risk appetite using two mirror symmetric distributions. For the explorative behavior (high risk appetite) we use a beta distribution of negative parameter range (β∈[−5, −2]), while for the exploitative behavior (low risk appetite) we use its symmetric positive parameter range (β∈[2, 5]). We further model a neutral risk level to be bounded within a probability distribution of β = 2.
6. **Homophily threshold (θ)**: The homophily threshold determines the extent to which people are willing to accept working with others based on their in-between attribute cosine similarity. Since we use four dimensions to determine workers' similarity (knowledge domain, nationality, educational level, age), we bound the homophily threshold within the range [1, 4]. The workers' default homophily threshold is set to 2.8 meaning that any similarity below this value is not considered sufficient to form a match.
7. **Teamwork quality coefficients (π**, **μ)**: The coefficients π and μ represent the weights attributed to team skill and interpersonal compatibility respectively. The default values are set to 0.4 for both π and μ. While we use these coefficients to adjust the weights of team skill and interpersonal compatibility, the same weight is taken off from the team size (1 - π−μ)^2^.

## 4. Results

In Section 4.1, we compare teamwork quality across the four models: Hive, C-SOT, R-SOT, and HiveHybrid and address the first research question (**RQ1: How does bottom-up team formation compare with top-down and hybrid approaches?**). Next, we address the second research question (**RQ2: How do population behavioral tendencies affect the outcome of bottom-up online teamwork?**) in three Sections, one for each of RQ2 sub question: Sections 4.2 and 4.3 examine teamwork quality according to changes in the workers' risk appetite and population size distributions, respectively, while The descriptive statistics report the mean and standard deviation (sd) of the model's teamwork quality. The standard deviation indicates the average amount of variability within a set of experiments. For example, *mean* = 0.716 and *sd* = 0.024 of the R-SOT model's best quality indicate, respectively, the mean and the standard deviation of the best teamwork gathered from thirty independent batch runs, as explained in the Methodology (Section 3.4).

### 4.1. Comparing Models: Radical Bottom-Up Yields the Highest and Lowest Teamwork Quality

**Figure 2:**
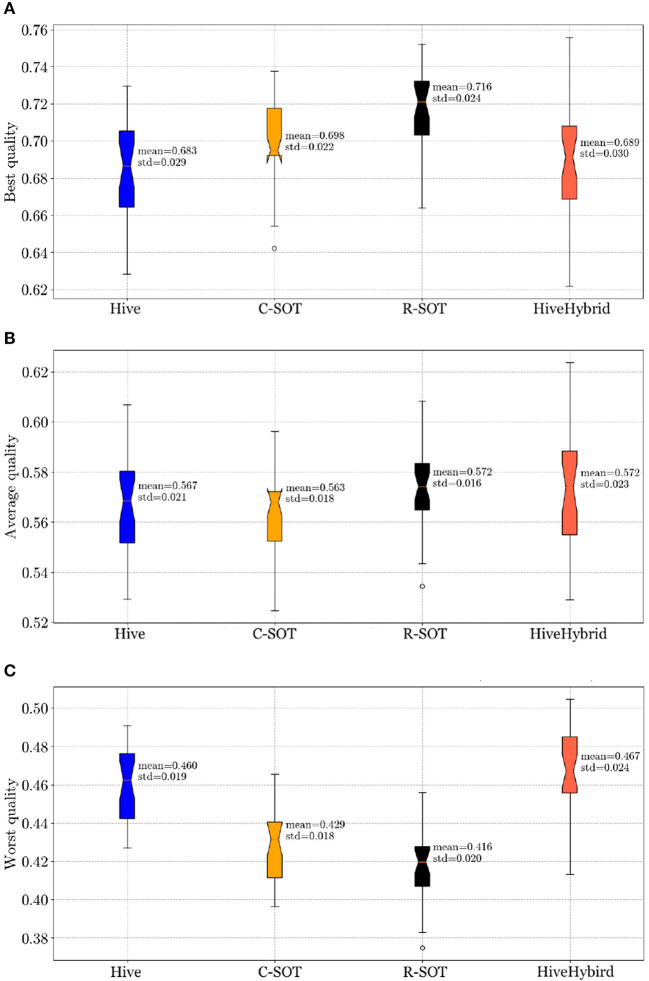
**Teamwork quality comparison** across four models: Hive, C-SOT, R-SOT, HiveHybrid. The boxplot displays the mean, standard deviation, and standard error of the teamwork quality. Overall, the best teamwork quality (Equation 2) belongs to the bottom-up models R-SOT (mean = 0.716) and C-SOT (mean = 0.698) followed by hybrid (mean = 0.689) and top-down (mean = 0.683). The average performance is fairly equal between models, with HiveHybrid and R-SOT having a slightly higher mean (mean = 0.572). The worst teamwork quality comes from the bottom-up models (R-SOT mean = 0.416, C-SOT mean = 0.429), followed by Hive (mean = 0.460). HiveHybrid performs the best at forming the least worst teamwork quality (mean = 0.467). **(A)** Best teamwork quality for Hive, C-SOT, R-SOT, and HiveHybrid. **(B)** Average teamwork quality for Hive, C-SOT, R-SOT, and HiveHybrid. **(C)** Worst teamwork quality for Hive, C-SOT, R-SOT, and HiveHybrid.

**Figure 3:**
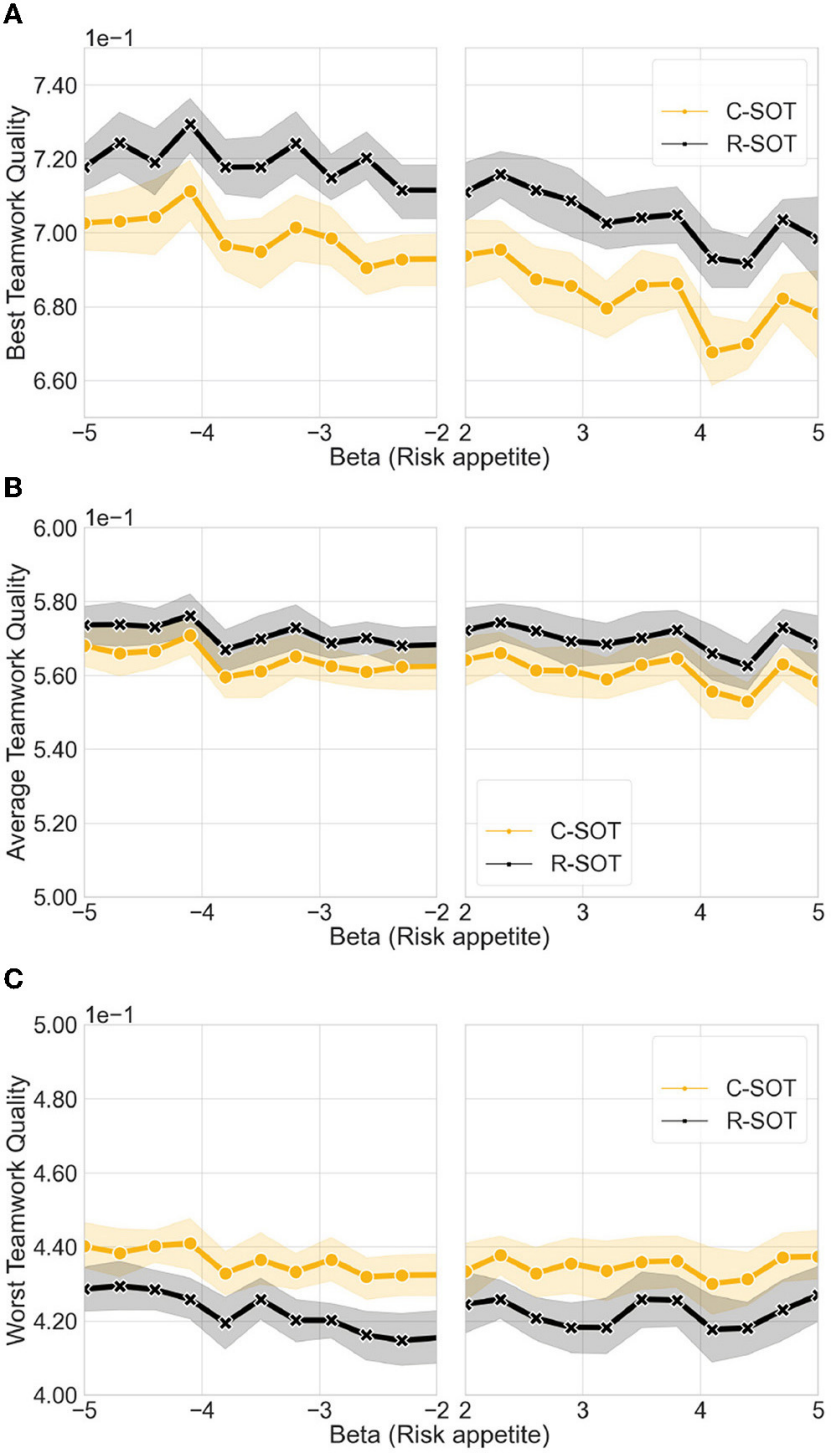
Comparison of the best and worst teamwork quality between bottom-up models, namely C-SOT and R-SOT, with **varying risk appetite levels**. The x axis illustrates the different risk levels generated according to two mirroring beta distributions: negative values (β ∈[−5, −2]) illustrate an exploratory, risk-prone user behavior (the more negative the more risk-prone); positive values (β ∈[2, 5]) illustrate an exploitative, risk-averse behavior (the more positive the more risk-averse). We observe that the best teamwork quality is affected by risk appetite, and that it decreases for both models as the users' willingness to change teams decreases. The average and worst teamwork quality remain unaffected by changes in the user population's risk levels. **(A)** Best teamwork quality for C-SOT and R-SOT with different risk appetite levels. **(B)** Average teamwork quality for C-SOT and R-SOT with different risk appetite levels. **(C)** Worst teamwork quality for C-SOT and R-SOT with different risk appetite levels.

**Figure 4:**
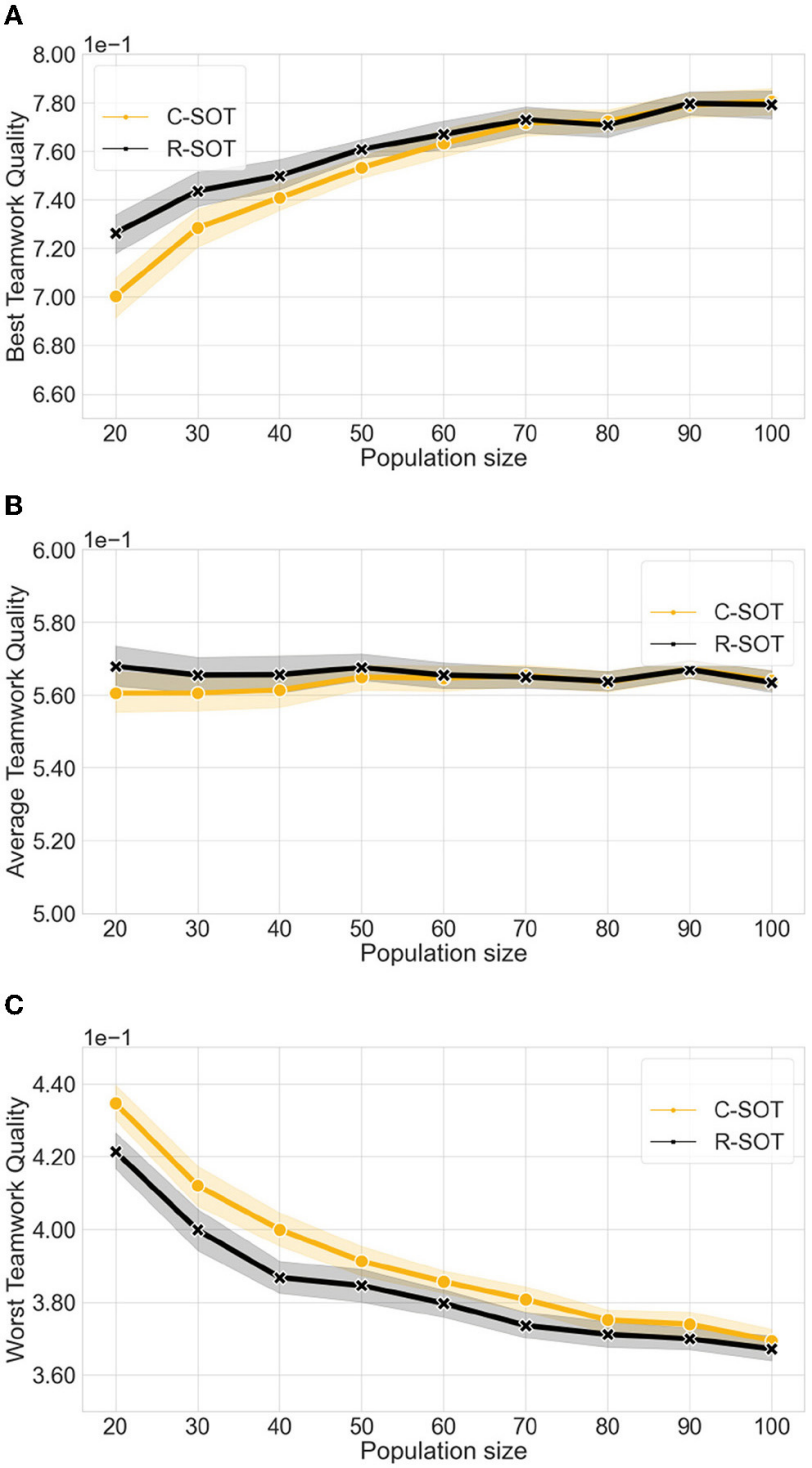
Comparison of the best and worst teamwork quality between two bottom-up models (C-SOT and R-SOT) with different population sizes. The x axes show the different simulated population sizes per hackathon in the ∈[20, 100] range. We observe that the best teamwork quality for both bottom-up models improves as the population grows from 20 to 90 individuals, and workers have more choice of teammates, reaching stability with populations of more than 90 and maintaining a best teamwork quality of ≈0.77 in both models. However, the worse teamwork quality also decreases steadily in both models lowering from ≈0.43 to ≈0.37 as the population grows indicating that large populations are not always beneficial to the performance of all teams. **(A) Best teamwork quality** for C-SOT and R-SOT with different **population sizes**. **(B) Average teamwork quality** for C-SOT and R-SOT with different **population sizes**. **(C) Worst teamwork quality** for C-SOT and R-SOT with different **population sizes**.

**Figure 5:**
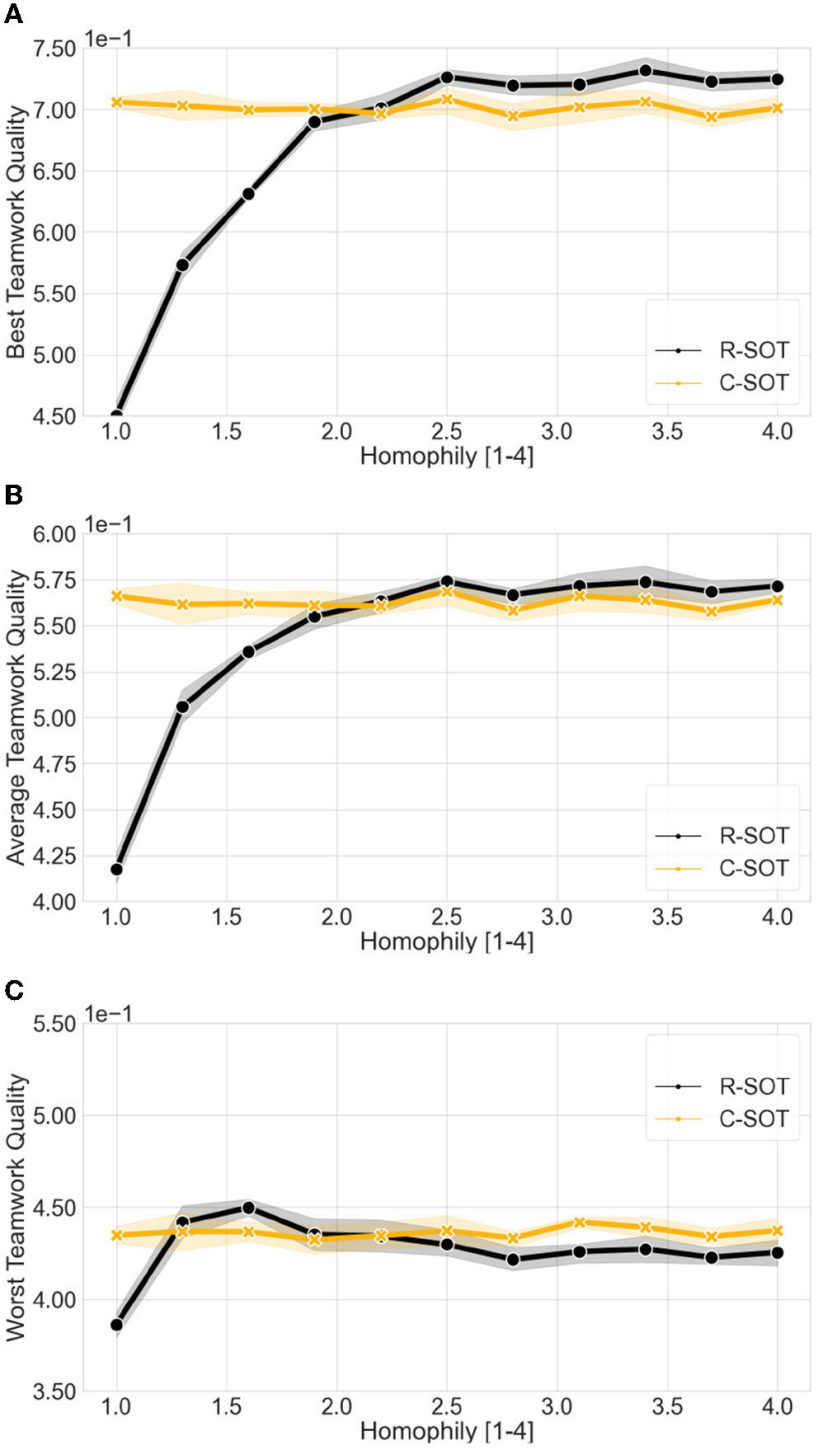
Comparison of the best and worst teamwork quality between bottom-up models (C-SOT and R-SOT) with different homophily thresholds. The x axes show the workers' different homophily levels (θ∈[1, 4]) where the lowest θ value represents the highest possible tolerance toward differences between workers' attributes, and the highest θ value the lowest tolerance. Even though the best teamwork quality for the two models improves as the homophily threshold grows, particularly with thresholds θ>2, the worst teamwork quality remains overall stable with the exception of R-SOT peaking around a threshold θ≈1.5 before settling around a worst teamwork quality of 0.42 with θ> 2.5. **(A) Best teamwork quality** for C-SOT and R-SOT with different **homophily (preference of working with similar teammates) thresholds (θ∈[1, 4])**. **(B) Average teamwork quality** for C-SOT and R-SOT with different **homophily thresholds (θ∈[1, 4])**. **(C) Worst teamwork quality** for C-SOT and R-SOT with different **homophily (preference of working with similar teammates) thresholds (θ∈[1, 4])**.

In this Section we address the first research question, namely **RQ1: How does bottom-up team formation compare with top-down and hybrid approaches?** Figures 2–5 shows the results of running a comparative study with all four models (R-SOT, C-SOT, Hive, and HiveHybrid) and utilizing the parameters stated in Section 3.4. We analyse the results below.

#### 4.1.1. Best Teamwork Quality

R-SOT has the highest average best quality (*mean=0.716, sd=0.024*), followed by C-SOT (*mean = 0.698, sd = 0.022*), HiveHybrid (*mean = 0.689, sd = 0.030*), and Hive (*mean = 0.683, sd = 0.029*) indicating that bottom-up models outperform the rest in forming the most competitive teams. Although standard deviations are relatively close across models, the standard error is greater in HiveHybrid than all other models possibly due to the unpredictability of combining suggested changes from the top-down community-based model with workers' decision.

#### 4.1.2. Average Teamwork Quality

R-SOT still performs better than the rest, although its mean is only marginally higher than the other models (*mean = 0.572, sd = 0.016*) followed by HiveHybrid (*mean = 0.572, sd = 0.023*), Hive (*mean = 0.567, sd = 0.021*), and C-SOT (*mean = 0.563, sd = 0.018*). In this comparison analysis, standard deviations are fairly close, while the standard error of HiveHybrid remains, by far, the largest in this comparison of the average teamwork quality. In fact, HiveHybrid's large standard error is present in all evaluations of teamwork quality.

#### 4.1.3. Worst Teamwork Quality

HiveHybrid has the least worst teamwork quality as its mean is above all others (*mean = 0.467, sd = 0.024*), followed by Hive (*mean = 0.460, sd = 0.019*), C-SOT (*mean=0.429, sd = 0.018*), and R-SOT (*mean = 0.416, sd = 0.020*). These final results indicate that the hybrid model is efficient at reducing the segregating patterns present in bottom-up systems, which lead to great variations of teamwork quality. Although the standard deviations are fairly close across models, HiveHybrid retains the largest standard error making it less consistent in its team formation.

#### 4.1.4. Statistical Analysis: R-SOT Outperforms in the Best and Loses at the Worst Teamwork Quality

Running a one-way ANOVA test on the results from the comparison of the teamwork quality between the four models we find the following.

- **Best teamwork quality:** The best teamwork quality is statistically significant between groups [*F*_(3, 116)_ = 10.477, *p* < 0.001]. Specifically, R-SOT performed significantly better than C-SOT (*p*=0.003), Hive (*p* <0.001), and hybrid Hive (*p*=0.001).
- **Average teamwork quality:** No statistical difference was found between models when comparing their average teamwork qualities [*F*_(3, 116)_ = 1.394, *p* < 0.248].
- **Worst teamwork quality:** We found statistically significant results between groups with the Worst teamwork quality. [*F*_(3, 116)_ = 35.122, *p* < 0.001]. Here, R-SOT performed significantly worst than C-SOT (*p* = 0.036), Hive (*p* < 0.001), and hybrid Hive (*p* < 0.001). The R-SOT model also performed significantly poorly compared to Hive (*p* < 0.001), and hybrid Hive (*p* < 0.001). Lastly, Hive and hybrid Hive did not differ significantly.

### 4.2. High Levels of Risk Appetite Segregate Teamwork Quality in Bottom-Up Models

This Section, combined with the two Sections that follow, dives deeper into the performance of bottom-up large-scale collaboration, and contributes to answering the second research question **RQ2: How do population behavioral tendencies affect the outcome of bottom-up online teamwork?** Specifically, it deals with the sub-question **RQ2.1: How do different risk appetites affect teamwork output in bottom-up models?** Looking at the highest teamwork quality (Figure 3) results across the two bottom-up models (R-SOT and C-SOT) and controlling for levels of the β value of the risk appetite distribution in the population, we note the following.

#### 4.2.1. Best Teamwork Quality

The radical self-organization approach (R-SOT) achieves better results in terms of the best teamwork quality (*mean* = 0.711, *std*=7.42e-3) compared to the conservative self-organized approach (C-SOT) (*mean* = 0.691, *std*=8.99e-3). This result indicates that the overall risk level of a population directly affects the workers' chances of forming optimal teams in bottom-up team formation strategies. Furthermore, high levels of risk appetite within a crowd population seem to be particularly beneficial to systems advocating radical changes in team structure. By lowering the overall risk appetite levels in both R-SOT and C-SOT, the performance of the best teamwork quality progressively suffers, dropping from 0.72 to 0.70 for R-SOT, and from 0.71 to 0.68 for C-SOT.

#### 4.2.2. Average Teamwork Quality

Results for the average teamwork quality are similar across both bottom-up models with R-SOT (*mean* = 0.569, *std*=2.16e-3) and C-SOT (*mean* = 0.562, *std*=3.03e-3) sharing similar outputs. These results indicate that the risk levels do not necessarily affect average performance despite of which bottom-up team formation strategy is used.

#### 4.2.3. Worst Teamwork Quality

Although the radical self-organized approach performed the highest when it came to the best teamwork quality, we observe that this approach is also the one that performs the worst from the two models R-SOT (*mean* = 0.422, *std*=2.90e-3) compared to C-SOT (*mean* = 0.435, *std*=2.43e-3). This result indicates that radically bottom-up approaches may inadvertently exacerbate the differences between teams, with the best workers choosing to team up with the best workers, leaving many of the average or low-performing workers behind, and causing segregated quality outputs.

### 4.3. Large Populations Strengthen Strong Teams in Bottom-Up Models

This Section answers the sub-question: **RQ2.2: How do different population sizes affect teamwork output in bottom-up models?** In the basic experimental setting (Section 3.4), we used a population size of 20 workers, which is a size that guarantees that workers can process the information concerning all other candidate co-workers effectively. However, the population size is a factor that can critically affect performance, as it is known to affect the worker collective's coordination costs. A larger population means a larger search space of available candidate teammates, and therefore more effort needed by the workers to process suitable teammates (Kittur and Kraut, 2008). We simulate nine separate and increasing population sizes starting from 20 (our basic simulation setting) and going up to 100 workers per pool to observe how the average best, worse, and median teamwork quality vary accordingly.

#### 4.3.1. Best Teamwork Quality

With an incremental growth in population size, both R-SOT and C-SOT improve their best performance shifting from an average best teamwork quality of 0.70 to one of 0.78. This result shows that bottom-up approaches particularly benefit from large scale participation as they rely on the diversity of workers' backgrounds and skills to form optimal teams.

#### 4.3.2. Average Teamwork Quality

As also observed in the previous Section for the parameter of risk appetite, we observe that the average teamwork quality neither benefits nor deteriorates from changes in population size and it remains relatively constant around 0.568.

#### 4.3.3. Worst Teamwork Quality

Lastly, the worst quality of bottom-up approaches drops from an average of 0.43 to an average of 0.36. The worst quality of R-SOT (*mean=*0.416) is indeed worse than C-SOT (*mean=* 0.429). This result may be explained by the fact that the R-SOT strategy dismantles teams having at least one unsatisfied worker and gives access to many more available workers of attractive attributes who can therefore settle for higher payoffs in the next round. Similarly to the results of the previous Section, here too we observe that the radical model (R-SOT) is the one yielding the highest best and the lowest worst quality.

### 4.4. Similar Workers Produce Higher Teamwork Quality in Bottom-Up Models

In this section, we address the last sub-question **RQ2.3: How does homophily, i.e., the tendency to prefer working with similar teammates, affect teamwork output in bottom-up models?** The homophily threshold determines the workers' tolerance to the diversity of attributes in others. Setting low homophily thresholds allows workers in the simulation to form larger teams since they are more open toward diverse team members. Using higher homophily thresholds pushes workers to carefully choose their teammates and only be interested in those who are most similar. Since there are four similarity attributes in the calculation of the cosine similarity score (knowledge domain, nationality, educational level, age), we use a homophily range of [1, 4] with a step of 0.3 allowing us to test ten variations of the threshold search space.

#### 4.4.1. Best Teamwork Quality

Testing all simulated thresholds we observe that the best teamwork quality of C-SOT is not affected by changes in homophily. However, R-SOT's best teamwork quality rapidly grows as the threshold increases from 0.50 with θ = 1.0 to 0.70 with θ = 2.8. After this growth, the best teamwork quality of the R-SOT model stabilizes and does not improve.

#### 4.4.2. Average Teamwork Quality

Similarly, the average teamwork quality is not significantly affected by changes in homophily in the C-SOT model while the R-SOT's average teamwork quality grows quite rapidly from 0.44 to 0.57 (from θ = 1 to θ = 1.5), and continues to rise before stabilizing around 0.72 with θ > 2.

#### 4.4.3. Worst Teamwork Quality

The worst teamwork quality is not affected by changes in homophily threshold for the C-SOT with the worst teamwork quality remaining stable at around 0.44. R-SOT's worst teamwork quality is more drastically affected by an increase in the homophily threshold, with an increase in quality between θ = 1.0 and θ = 1.6 and then a gradual decrease and stabilization after θ = 2.5. This is a similar pattern (sharp rise and then stabilization) like the one we saw R-SOT following in the best and average teamwork quality results, albeit with less intensity as we go from best to worst quality.

## 5. Discussion

### 5.1. Bottom-Up Models Are Ideal for Large Scale Crowdsourcing Collaborative Innovation

We observe that the bottom-up models R-SOT and C-SOT are more effective than the top-down (Hive) and hybrid solutions (HiveHybrid) at forming teams with the best teamwork quality. In these self-organizing systems, workers seek collaborators based on how likely they are to explore the search space and how tolerant they are toward diverse teammates. Whether workers will explore further teammates depends on the reward they received with their old team, bounded by their risk appetite. For example, low-risk workers will keep working with the same teammate even if they did not get a high reward, while high-risk workers will change more frequently. Although skill is therefore not explicitly present in the worker's search function, since it is a latent feature that workers cannot directly have access to concerning their teammates, we observe that *gradually workers discover their in-between skills by implicitly evaluating the results of their existing collaborations against others through the rewards each team received*.

This result shows that *bottom-up systems lift the requirement for intensive skill profiling before they can make good team formation decisions*, and it is important for platforms for multiple reasons. First, it renders bottom-up models *more appropriate for innovation-related tasks* for which the exact skills that will be needed to solve the task are not easily measurable or even known a priori to the collaborative platform (Gerber et al., 1999). Second, by lifting the requirement for designing tailor-made profiling tests and team formation algorithms, *bottom-up models are more cost-effective than their top-down or hybrid counterparts*, and are also particularly useful for introducing new tasks in the platform for which no profile information concerning worker competencies or matching mechanism is yet present. One important point to make here is that these advantages of bottom-up models refer to the best teamwork quality achieved, but that, at the same time, these models also tend to segregate the worst-performing teams. In other words, *bottom-up models help form principally strong and competitive coalitions which may form at the expense of other teams*. Therefore, self-organization could be more appropriate and cost-effective for commercial platforms targeting at the competitive production of tasks, rather than tasks for which “no worker is left behind” (e.g., in educational settings). Overall, our comparative analysis of team formation system models in crowdsourcing collaborative innovation indicates that platforms can “trust the crowd” to form teams as long as they favor competition over cooperation and as long as they prefer competitive teams over a centralized re-distribution of social capital.

### 5.2. Hybrid Systems Are Best for Semi-centralized Social Capital Redistribution

As we have seen, bottom-up systems are the best at producing teams of the highest teamwork quality. However, having the best team is not the sole representative metric of collective performance in social cooperative scenarios. In fact, in the case of our bottom-up models, the worse-off teams do not seem to benefit from a self-organizing system as their teamwork quality notably suffers compared to the worst teams from algorithmic-mediated team formation solutions. Especially, *the hybrid approach HiveHybrid is the most effective at bridging the gap between best and worst teamwork qualities* forming teams that are closer in the way they perform. This ability to redistribute resources among a population to help all teams achieve similar teamwork quality is exceptionally favorable in settings where global objectives are of equal importance to local interactions as it is in the case of groups of learners. Massive Open Online Courses (MOOCs) are an example of large-scale collaborative settings that would benefit from fair semi-centralized clustering to allow all learners to partake in useful and educational teamwork. It could also help with reducing drop-outs as learners would be motivated to partake in teams from which they can learn and share knowledge. Through hybrid approaches to team formation, online learners would be given the final decision over algorithmic prompts carrying decisive advantages over fully top-down implementations that typically disregard individual preferences.

Control of social capital and resource redistribution is not the only advantage of hybrid systems. Connecting workers' decisions—which are by definition local and discrete—with global utility and centralized coordination *could help with situations where workers can no longer process information by themselves*. Often with online crowdsourcing innovation projects, several hundred individuals take part in events and seek collaborators. As much as the team formation system relies on their ability to self-organize to produce optimal teamwork outcomes, there is always the looming risk that workers cannot assess more than a limited amount of possible collaborators at a time. This means that workers are likely to miss out on potentially better matches as they simply cannot have a comprehensive view of all options unless they scout the entire pool of workers. However, with prolonged exploration workers do not have the time to settle into teams and may discard ideal collaborators to continue their search. Due to this extraneous cognitive overload and excessive search space, *a system that can fittingly combine algorithmic mediation and worker agency—such as HiveHybrid—could be a more suitable alternative to fully self-organized and fully top-down systems even though they may be less effective at forming highly competitive teams*.

### 5.3. Generalizing to Other Collaborative Settings

In this study, we simulated an open collaboration scenario where crowds gather to collaborate on a complex problem. We chose a hackathon as an example of a design-sprint-like event for which crowds compete for prizes while collaborating in teams. As in reality, our simulations represent workers forming project teams either through top-down mediation (upon organizers' decision) or bottom-up negotiation (workers choose their teammates and self-organize). Although traditionally confined to software development, hackathons have developed to serve other scopes, for example, by hosting charity events, public memorials, professional networking, and more, and are therefore much broader than their cryptography development ancestor (Briscoe, 2014). For this reason, hackathons can be used as general-purpose initiatives to attract crowd participation and gather expertise and innovation. Online hackathons have also become attractive mediums for the involvement of citizen crowds in decision-making processes (Temiz, 2021). For example, “Hack The Crisis” is, to date, the most popular crowdsourced global movement connecting crowds to solve complex societal challenges such as pandemics prevention and emergency response (Hack the Crisis Team, 2021). The chosen setting could be applied to large-scale crowd empowerment through open challenges, open education, and social impact.

Regarding the components of the simulation, we modeled only an abstract set of worker skills especially since some hackathons' organizers filter attendance based on functional background and expertise with the intent to harvest specialized knowledge from the crowd. However, the worker model could be easily expanded to other tasks and settings. For example, in a scenario where students form teams, their attributes would represent interests, preferences, and abilities instead of the functional background, personality, and skill as we modeled in this study. Furthermore, some hackathons are characterized by rounds of sprints which we have devoted to individual/algorithmic decision-making and search space. Moreover, in real-life hackathons, it is not unusual that these rounds provide organizers regular opportunities to monitor and evaluate teamwork as the event unfolds. In our study, we use the same concept to evaluate teamwork quality and to allow workers (and algorithms) to rotate teams. From MOOCs to citizen science, these elements of the system can be adjusted to correspond with periods of recollection and assessment that are often present in large-scale crowdsourcing activities. Finally, hackathons usually end up with a selection of the best projects and the best teams, which, in this case, is the main metric for assessing the adequacy of the team formation system models. Generalizing this setting to other scenarios, we suggest that the evaluation could be adjusted to whichever factor the event organizer wishes to assess (e.g., communication, coordination, the balance of contribution and effort, etc.). For example, teams of learners will be likely evaluated based on mutual support, cohesion, and effort, thus differing from software development teams focused on product quality, team efficiency, and profitability.

## 6. Limitations

In this section, we list and discuss four main system design choices that could be improved or modified in future studies as follows.

Modeling worker attributes and recruitment through AMT may not be comparable to other platforms. In this paper, we have used AMT as the platform of reference for modeling worker demographic attributes (Difallah et al., 2015; Lykourentzou et al., 2016a) and recruitment. This choice allowed us to ascertain a degree of reliability and applicability since the demographic distribution adhered to existing statistical records. However, as crowdsourcing platforms evolve and differentiate, many more platforms offer like-minded individuals ways to collaborate and participate in disparate projects. The most prevalent crowd population on platforms facilitating creative tasks, such as OpenIDEO, Upwork, Fiverr, or even creative hubs, may have different demographic attributes than their AMT counterpart, which is mainly used to serve micro-tasks. We strongly encourage future studies to consider additional platforms of reference to model workers' profiles (such as educational level, personality, and age) and recruitment, which could be more relevant to creative hackathons and complex problem-solving.

### 6.1. Homophily Threshold Modeled on the Entire Population

Unlike worker risk appetite, homophily was modeled on the whole population as a shared threshold rather than on an individual-to-individual case through a non-uniform probability distribution. This modeling choice also means that it is not possible to identify how individual homophily might have affected the behavior of a worker in a pool with diverse homophily attitudes. In future studies, modeling the personal preferences of collaborators would help to fine-grain our assessment of the impact of homophily in team formation. It would also help with evaluating how different attitudes toward diversity combined affect the formation of more or less stable teams and to what extent it influences teamwork quality.

### 6.2. Risk Appetite Goes Beyond the Tendency to Explore Collaborators

In our model of the workers, we attributed risk appetite to the individual tendency to explore novel collaboration. In this context, risk appetite can be thought of as a behavioral property encompassing one's curiosity and extroversion. Nonetheless, risk appetite could also determine one's preference for a particular task and ways of executing it. Modeling task choices, task execution, and effort as part of the workers' risk appetite would also determine their stress and energy levels and delineate a finer-grained representation of human behavior (Chiang et al., 2021). We, therefore, suggest extending the significance and functionality of the risk appetite attribute in future simulations.

### 6.3. Sensitivity Analysis Limited to Bottom-Up Models

After comparing the four models (C-SOT, R-SOT, Hive, and HiveHybrid) on a set of specific parameters, we have systematically varied the parameters of risk appetite, population size, and homophily threshold for the comparison of the bottom-up models. This analysis permitted us to examine in detail the models' response to varying population behavioral patterns, across the three aforementioned worker attributes. For our main scenario we have chosen a specific and fixed set of parameters; although these modeling choices have been based on the literature, they do limit the applicability of our results to the specific population characteristics. Performing a systematic sensitivity analysis for the main scenario can, in the future, permit to examine whether the current results can be generalized to scenarios with other demographics or whether there are any mixed effects, for instance between the team size and the workers' homophily threshold.

### 6.4. Teamwork Quality Function Limited in Scope

Our evaluation of teamwork quality is based on the assumption that certain attributes together matter most in determining the probability of success of a team. We identified team skill, interpersonal compatibility, and team size as the determining factors. Although these factors have been shown in the literature to critically affect teamwork performance, there may be additional aspects of the collaboration that also play a significant role depending on the real-world task at-hand. For example, communication quality, the ability to think out-of-the-box as a team may also affect the final result. Follow-up studies could therefore examine additional quality metrics and even evaluate different methods for calculating teamwork quality than the one used in this study.

### 6.5. Worker Search Space Unhindered by Cognitive and Temporal Constraints

Another assumption present in this study is that workers are not constrained in their search of available teammates. This means that if a large pool of workers is available, workers can evaluate all possible team combinations and pick the one with the highest utility. In real-life settings, this is not always possible as information may be missing and time and resources may be lacking to carry out a thorough evaluation of this kind. Future simulations should take into account the limitations that workers may face when assessing others, especially as the size of the worker pool increases, and convey these in their definition of the search function. It is possible that workers can truly only process a few candidates at a time, and their judgment can be affected by presentation or popularity biases.

### 6.6. Hive Is One of Many Kinds of Top-Down Models

Our comparison of different team formation models uses only one top-down approach, namely Hive. Although the Hive algorithm is a latest state-of-the-art community-based solution, it does not represent many other kinds of top-down approaches. Due to this limitation, our comparison cannot be entirely generalized to other top-down team formation systems, aside from the acknowledgement that they do not grant worker agency in decision-making. Testing other approaches, such as bi-partite graphs and stable matching algorithms will give future studies more comprehensive knowledge of the effectiveness of these approaches in collaborative crowdsourcing scenarios and how they compare to self-organized and hybrid solutions.

## 7. Conclusion

With the rapid growth of crowdsourcing platforms used for collaborative innovation generation and citizen participation, team formation among members of a crowd becomes increasingly pertinent. This study evaluates how different approaches to crowdsourcing team formation impact teamwork through bottom-up, top-down, and hybrid models. Using a simulated hackathon scenario, we gathered results from the collaboration between strategic worker agents showing that bottom-up models are convincingly more effective at forming highly competitive teams but do not succeed at redistributing equally resources within the crowd population. On the contrary, the hybrid system which combines bottom-up worker agency with top-down algorithmic mediation bridged this gap by forming teams of closer teamwork quality. The purely top-down approach performed averagely whilst still limiting worker agency in team formation. We further observe that high-risk appetite levels, large population sizes, and high homophily thresholds of the involved crowd worker population positively affect teamwork quality in bottom-up approaches. This study furthers our assessment of the impact of self-organization in large-scale collaborative crowd innovation and helps the design of systems incorporating agency in algorithmic mediation in team formation.

## Data Availability Statement

The code supporting this article is available as Open Source in: https://github.com/ilykour/self_organised_teams.

## Author Contributions

All authors contributed to the article and approved the submitted version.

## Conflict of Interest

The authors declare that the research was conducted in the absence of any commercial or financial relationships that could be construed as a potential conflict of interest.

## Publisher's Note

All claims expressed in this article are solely those of the authors and do not necessarily represent those of their affiliated organizations, or those of the publisher, the editors and the reviewers. Any product that may be evaluated in this article, or claim that may be made by its manufacturer, is not guaranteed or endorsed by the publisher.

## Footnotes

^1^For this study, we chose Amazon Mechanical Turk as the example hiring platform since its demographics are well-known (Difallah et al., 2018). Nonetheless, we acknowledge that the population is expected to differ on platforms such as Upwork and other virtual creative hubs.

^2^This applies less weight to that factor.

## References

[B1] Ahmed F. Dickerson J. Fuge M. (2020). Forming diverse teams from sequentially arriving people. J. Mech. Des. 142:111401. 10.1115/1.4046998

[B2] Ananny M. (2016). Toward an ethics of algorithms: convening, observation, probability, and timeliness. Sci. Technol. Hum. Values 41, 93–117. 10.1177/0162243915606523

[B3] Anzola D. Barbrook-Johnson P. Cano J. I. (2017). Self-organization and social science. Comput. Math. Organ. Theory 23, 221–257. 10.1007/s10588-016-9224-2

[B4] Avis D. (1983). A survey of heuristics for the weighted matching problem. Networks 13, 475–493. 10.1002/net.3230130404

[B5] Barnes S.-A. Green A. De Hoyos M. (2015). Crowdsourcing and work: individual factors and circumstances influencing employability. New Technol. Work Employ. 30, 16–31. 10.1111/ntwe.12043

[B6] Berg J. (2015). Income security in the on-demand economy: findings and policy lessons from a survey of crowdworkers. Comp. Lab. L. & Pol'y J. 37, 543.

[B7] Berger-Tal O. Nathan J. Meron E. Saltz D. (2014). The exploration-exploitation dilemma: a multidisciplinary framework. PLoS ONE 9,e95693. 10.1371/journal.pone.0095693 24756026 PMC3995763

[B8] Betts A. Bloom L. (2014). Humanitarian Innovation: The State of the Art. United Nations Office for the Coordination of Humanitarian Affairs (OCHA).

[B9] Briscoe G. (2014). Digital Innova: The Hackathon Phenomenon.

[B10] Carless S. A. De Paola C. (2000). The measurement of cohesion in work teams. Small Group Res. 31, 71–88. 10.1177/10464964000310010430672820

[B11] Centola D. Gonzalez-Avella J. C. Eguiluz V. M. San Miguel M. (2007). Homophily, cultural drift, and the co-evolution of cultural groups. J. Confl. Resolut. 51, 905–929. 10.1177/0022002707307632

[B12] Chiang C.-E. Chen Y.-C. Lin F.-Y. Feng F. Wu H.-A. Lee H.-P.. (2021). “‘I got some free time': investigating task-execution and task-effort metrics in mobile crowdsourcing tasks,” in Proceedings of the 2021 CHI Conference on Human Factors in Computing Systems (Yokohama), 1–14. 10.1145/3411764.3445477

[B13] Costa A. C. Fulmer C. A. Anderson N. R. (2018). Trust in work teams: an integrative review, multilevel model, and future directions. J. Organ. Behav. 39, 169–184. 10.1002/job.2213

[B14] De Dreu C. K. West M. A. (2001). Minority dissent and team innovation: the importance of participation in decision making. J. Appl. Psychol. 86,1191. 10.1037/0021-9010.86.6.1191 11768061

[B15] De Stefano V. (2015). The rise of the just-in-time workforce: on-demand work, crowdwork, and labor protection in the gig-economy. Comp. Lab. L. & Pol'y J. 37,471. 10.2139/ssrn.2682602

[B16] Degli Antoni G. Fia M. Sacconi L. (2021). Specific investments, cognitive resources, and specialized nature of research production in academic institutions: why shared governance matters for performance. J. Instit. Econ. 18, 1–22. 10.1017/S1744137421000655

[B17] Deitz C. (2016). Pragmatism and mechanical Turk: citizenship and labor rights in digital communities of knowledge. J. Media Ethics 31, 264–266. 10.1080/23736992.2016.1228816

[B18] Difallah D. Filatova E. Ipeirotis P. (2018). “Demographics and dynamics of mechanical Turk workers,” in Proceedings of the Eleventh ACM International Conference on Web Search and Data Mining (Los Angeles, CA), 135–143. 10.1145/3159652.3159661

[B19] Difallah D. E. Catasta M. Demartini G. Ipeirotis P. G. Cudré-Mauroux P. (2015). “The dynamics of micro-task crowdsourcing: the case of amazon mTurk,” in Proceedings of the 24th International Conference on World Wide Web (Florence), 238–247. 10.1145/2740908.2744109

[B20] Eugene N. Lee C. Famoye F. (2002). Beta-normal distribution and its applications. Commun. Stat. Theory Methods 31, 497–512. 10.1081/STA-120003130

[B21] Faraj S. Pachidi S. Sayegh K. (2018). Working and organizing in the age of the learning algorithm. Inform. Organ. 28, 62–70. 10.1016/j.infoandorg.2018.02.005

[B22] Florisson R. Mandl I. (2018). “Platform work: Types and implications for work and employment-Literature review,” in European Foundation for the Improvement of Living and Working Conditions.

[B23] Furlow L. (2000). Job profiling: building a winning team using behavioral assessments. J. Nurs. Administr. 30, 107–111. 10.1097/00005110-200003000-00001 10725938

[B24] Gaikwad S. Morina D. Nistala R. Agarwal M. Cossette A. Bhanu R.. (2015). “DAEMO: a self-governed crowdsourcing marketplace,” in Adjunct Proceedings of the 28th Annual ACM Symposium on User Interface Software & Technology (Daegu), 101–102. 10.1145/2815585.2815739

[B25] Gaikwad S. N. S. Whiting M. E. Gamage D. Mullings C. A. Majeti D. Goyal S.. (2017). “The DAEMO crowdsourcing marketplace,” in Companion of the 2017 ACM Conference on Computer Supported Cooperative Work and Social Computing (Portland, OR), 1–4. 10.1145/3022198.3023270

[B26] Gerber C. Siekmann J. Vierke G. (1999). “Holonic multi-agent systems,” in Self-organising Software. Natural Computing Series, eds G. Di Marzo Serugendo, M. P. Gleizes, and A. Karageorgos (Berlin; Heidelberg: Springer). pp. 238–263.

[B27] Gilson L. L. and Shalley, C. E. (2004). A little creativity goes a long way: an examination of teams' engagement in creative processes. J. Manage. 30, 453–470. 10.1016/j.jm.2003.07.001

[B28] Gray M. L. Suri S. (2019). Ghost Work: How to Stop Silicon Valley From Building a New Global Underclass. New York, NY: Eamon Dolan Books.

[B29] Haas M. Mortensen M. (2016). The secrets of great teamwork. Harvard Bus. Rev. 94, 70–76. 27491197

[B30] Hack the Crisis Team. (2021). Hack The Crisis Join the Brightest Minds to Tackle COVID-19. Available online at: https://www.hackthecrisis.nl/en/#about (accessed September 17, 2021).

[B31] Haeussler C. Sauermann H. (2020). Division of labor in collaborative knowledge production: the role of team size and interdisciplinarity. Res. Policy 49,103987. 10.1016/j.respol.2020.103987

[B32] Hasteer N. Bansal A. Murthy B. (2015). An agent based simulation study of association amongst contestants in crowdsourcing software development through preferential attachment. J. Eng. Appl. Sci. 10, 2509–2517.

[B33] Haun D. Over H. (2015). “Like me: a homophily-based account of human culture,” in Epistemological Dimensions of Evolutionary Psychology, ed T. Breyer (New York, NY: Springer), 117–130. 10.1007/978-1-4939-1387-9_6

[B34] Highsmith J. (2009). Agile Project Management: Creating Innovative Products. Boston: Pearson education.

[B35] Jackson S. E. (1983). Participation in decision making as a strategy for reducing job-related strain. J. Appl. Psychol. 68,3. 10.1037/0021-9010.68.1.3

[B36] Jarrahi M. H. Sutherland W. Nelson S. B. Sawyer S. (2020). Platformic management, boundary resources for gig work, and worker autonomy. Comput. Support. Cooper. Work 29, 153–189. 10.1007/s10606-019-09368-7

[B37] Jiang J. An B. Jiang Y. Zhang C. Bu Z. Cao J. (2019). Group-oriented task allocation for crowdsourcing in social networks. IEEE Trans. Syst. Man Cybern. 51, 4417–4432. 10.1109/TSMC.2019.2933327

[B38] Juárez J. Santos C. Brizuela C. A. (2021). A comprehensive review and a taxonomy proposal of team formation problems. ACM Comput. Surveys 54, 1–33. 10.1145/3465399

[B39] Kenna R. Berche B. (2012). Managing research quality: critical mass and optimal academic research group size. IMA J. Manage. Math. 23, 195–207. 10.1093/imaman/dpr021

[B40] Khan V.-J. Papangelis K. Lykourentzou I. Markopoulos P. (2019). Macrotask Crowdsourcing. Cham: Springer International Publishing. 10.1007/978-3-030-12334-5

[B41] Kittur A. Kraut R. E. (2008). “Harnessing the wisdom of crowds in wikipedia: quality through coordination,” in Proceedings of the 2008 ACM Conference on Computer Supported Cooperative Work (San Diego, CA), 37–46. 10.1145/1460563.1460572

[B42] Lakhani K. R. Fayard A.-L. Levina N. Pokrywa S. H. (2012). OpenIDEO. Harvard Bus. Sch. Technol. Operat. Mgt. Unit Case. 612–666.

[B43] Lakhani K. R. Lonstein E. (2008). InnoCentive.com (A). Boston, MA: Harvard Business School case. 608, 170.

[B44] Latora V. Marchiori M. (2001). Efficient behavior of small-world networks. Phys. Rev. Lett. 87, 198701. 10.1103/PhysRevLett.87.198701 11690461

[B45] Lawler E. Worley C. (2009). Designing organizations that are built to change. Organ. Fut. 2, 188–202.

[B46] Liu Q. Luo T. Tang R. Bressan S. (2015). “An efficient and truthful pricing mechanism for team formation in crowdsourcing markets,” in 2015 IEEE International Conference on Communications (ICC), London: IEEE. 567–572. 10.1109/ICC.2015.7248382

[B47] LLP D. T. T. I. (2020). Future of Work Accelerated: Learnings From the Covid-19 Pandemic. Available online at: https://www2.deloitte.com/content/dam/Deloitte/in/Documents/human-capital/in-consulting-accelerated-hc-consulting-noexp.pdf (accessed April 1, 2022).

[B48] Lykourentzou I. Antoniou A. Naudet Y. Dow S. P. (2016a). “Personality matters: Balancing for personality types leads to better outcomes for crowd teams,” in Proceedings of the 19th ACM Conference on Computer-Supported Cooperative Work & Social Computing (San Francisco), 260–273. 10.1145/2818048.2819979

[B49] Lykourentzou I. Liapis A. Papastathis C. Papangelis K. Vassilakis C. (2019). “Exploring self-organisation in crowd teams,” in Conference on e-Business, e-Services and e-Society (Cham; Trondheim: Springer), 164–175. 10.1007/978-3-030-39634-3_15

[B50] Lykourentzou I. Papadaki K. Vergados D. J. Polemi D. Loumos V. (2010). Corpwiki: a self-regulating wiki to promote corporate collective intelligence through expert peer matching. Inform. Sci. 180, 18–38. 10.1016/j.ins.2009.08.003

[B51] Lykourentzou I. Vinella F. L. Ahmed F. Papastathis C. Papangelis K. Khan V.-J.. (2021). Self-organizing teams in online work settings. arXiv[Preprint]. arXiv:2102.07421. 10.48550/arXiv.2102.07421

[B52] Lykourentzou I. Wang S. Kraut R. E. Dow S. P. (2016b). “Team dating: a self-organized team formation strategy for collaborative crowdsourcing,” in Proceedings of the 2016 CHI Conference Extended Abstracts on Human Factors in Computing Systems (San Jose, CA), 1243–1249. 10.1145/2851581.2892421

[B53] Manyika J. Lund S. Bughin J. Robinson K. Mischke J. Mahajan D. (2016). Independent-Work-Choice-Necessity-and-the-Gig-Economy. Technical Report, McKinsey Global Institute.

[B54] Marston W. M. (2013). Emotions of Normal People. Oxon: Routledge. 10.4324/9781315010366

[B55] Martius G. Herrmann J. M. (2012). Variants of guided self-organization for robot control. Theory Biosci. 131, 129–137. 10.1007/s12064-011-0141-0 22116785

[B56] Marwell G. Oliver P. E. Prahl R. (1988). Social networks and collective action: a theory of the critical mass. III. Am. J. Sociol. 94, 502–534. 10.1086/229028

[B57] Marzo Serugendo G. D. Foukia N. Hassas S. Karageorgos A. Mostéfaoui S. K. Rana O. F.. (2003). “Self-organisation: paradigms and applications,” in International Workshop on Engineering Self-Organising Applications (Berlin; Heidelberg: Springer), 1–19. 10.1007/978-3-540-24701-2_1

[B58] McPherson M. Smith-Lovin L. Cook J. M. (2001). Birds of a feather: homophily in social networks. Annu. Rev. Social. 27, 415–444. 10.1146/annurev.soc.27.1.415

[B59] Moe N. B. Dingsøyr T. (2008). “Scrum and team effectiveness: theory and practice,” in International Conference on Agile Processes and Extreme Programming in Software Engineering (Berlin; Heidelberg: Springer), 11–20. Springer. 10.1007/978-3-540-68255-4_2

[B60] Monsef S. Javanmard S. H. Amini-Rarani M. Yarmohammadian M. H. Yazdi Y. Haghshenas A. (2021). Idea generation through Hackathon event in emergencies and disasters, with emphasis on managing flash flood disaster. Disast. Med. Publ. Health Prepared. 15, 1–5. 10.1017/dmp.2021.30 34002685

[B61] Moreland R. L. (2010). Are dyads really groups? Small Group Res. 41, 251–267. 10.1177/1046496409358618

[B62] Nolte A. Pe-Than E. P. P. Filippova A. Bird C. Scallen S. Herbsleb J. D. (2018). You hacked and now what? -exploring outcomes of a corporate Hackathon. Proc. ACM Hum. Comput. Interact. 2, 1–23. 10.1145/3274398

[B63] Nurzaman S. G. Yu X. Kim Y. Iida F. (2014). Guided self-organization in a dynamic embodied system based on attractor selection mechanism. Entropy 16, 2592–2610. 10.3390/e16052592

[B64] Ortu M. Destefanis G. Counsell S. Swift S. Tonelli R. Marchesi M. (2017). How diverse is your team? Investigating gender and nationality diversity in Github teams. J. Softw. Eng. Res. Dev. 5, 1–18. 10.1186/s40411-017-0044-y

[B65] Popescu G. H. Petrescu I. E. Sabie O. M. (2018). Algorithmic labor in the platform economy: digital infrastructures, job quality, and workplace surveillance. Econ. Manage. Financ. Mark. 13, 74–79. 10.22381/EMFM13320184

[B66] Prokopenko M. (2009). Guided Self-Organization. Taylor & Francis. 10.2976/1.3233933 PMC2801529 20057962

[B67] Prolific Team. (2021). Prolific Demographics of Participant Pool. Available online at: https://researcher-help.prolific.co/hc/en-gb/articles/360009391633-Exporting-Prolific-Demographic-Data (accessed September 21, 2021).

[B68] Rahman H. Roy S. B. Thirumuruganathan S. Amer-Yahia S. Das G. (2019). Optimized group formation for solving collaborative tasks. VLDB J. 28, 1–23. 10.1007/s00778-018-0516-7

[B69] Ramadi K. B. Nguyen F. T. (2021). Rapid crowdsourced innovation for covid-19 response and economic growth. NPJ Digit. Med. 4, 1–5. 10.1038/s41746-021-00397-5 33564061 PMC7873189

[B70] Rasmussen T. H. Jeppesen H. J. (2006). Teamwork and associated psychological factors: a review. Work Stress 20, 105–128. 10.1080/02678370600920262

[B71] Retelny D. Bernstein M. S. Valentine M. A. (2017). No workflow can ever be enough: How crowdsourcing workflows constrain complex work. Proc. ACM Hum. Comput. Interact. 1, 1–23. 10.1145/3134724

[B72] Retelny D. Robaszkiewicz S. To A. Lasecki W. S. Patel J. Rahmati N.. (2014). “Expert crowdsourcing with flash teams,” in Proceedings of the 27th Annual ACM Symposium on User Interface Software and Technology (Honolulu, HI), 75–85. 10.1145/2642918.2647409

[B73] Rokicki M. Zerr S. Siersdorfer S. (2015). “Groupsourcing: team competition designs for crowdsourcing,” in Proceedings of the 24th International Conference on World Wide Web (Florence), 906–915. 10.1145/2736277.2741097

[B74] Roy S. Balamurugan C. Gujar S. (2013). “Sustainable employment in India by crowdsourcing enterprise tasks,” in Proceedings of the 3rd ACM Symposium on Computing for Development (Bangalore), 1–2. 10.1145/2442882.2442904

[B75] Salehi N. Bernstein M. S. (2018). Hive: collective design through network rotation. Proc. ACM Hum. Comput. Interact. 2, 1–26. 10.1145/3274420

[B76] Schriner A. Oerther D. (2014). No really (crowd) work is the silver bullet. Proc. Eng. 78, 224–228. 10.1016/j.proeng.2014.07.060

[B77] Silberman M. S. Tomlinson B. LaPlante R. Ross J. Irani L. Zaldivar A. (2018). Responsible research with crowds: pay crowdworkers at least minimum wage. Commun. ACM 61, 39–41. 10.1145/3180492

[B78] Smith R. Leberstein S. (2015). Rights on Demand. Ensuring Workplace Standards and Worker Security In the On-Demand Economy. National Employment Law Project, Washington, DC.

[B79] Taha H. A. (2013). Operations-Research-An-Introduction-10th-Ed. Harlow.

[B80] Temiz S. (2021). Open innovation *via* crowdsourcing: a digital only Hackathon case study from Sweden. J. Open Innov. 7:39. 10.3390/joitmc7010039

[B81] Valentine M. A. Retelny D. To A. Rahmati N. Doshi T. Bernstein M. S. (2017). “Flash organizations: crowdsourcing complex work by structuring crowds as organizations,” in Proceedings of the 2017 CHI Conference on Human Factors in Computing Systems (New York, NY), 3523–3537. 10.1145/3025453.3025811

[B82] Wang R. (2020). Marginality and team building in collaborative crowdsourcing. Online Inform. Rev. 44, 827–846. 10.1108/OIR-09-2018-0269

[B83] Wang S. Yeoh W. Ren J. Lee A. (2021). Learnings and implications of virtual Hackathon. J. Comput. Inform. Syst. 62, 1–13. 10.1080/08874417.2020.1864679

[B84] Whiting M. E. Gamage D. Goyal S. Gilbee A. Majeti D. Richmond-Fuller A.. (2017). “Designing a constitution for a self-governing crowdsourcing marketplace,” in Collective Intelligence Conference (Brooklyn, NY), 15–16.

[B85] Whitson J. R. Simon B. Parker F. (2021). The missing producer: rethinking indie cultural production in terms of entrepreneurship, relational labour, and sustainability. Eur. J. Cult. Stud. 24, 606–627. 10.1177/1367549418810082 33867811 PMC8022774

[B86] Wood A. J. Graham M. Lehdonvirta V. Hjorth I. (2019). Networked but commodified: the (dis) embeddedness of digital labour in the gig economy. Sociology 53, 931–950. 10.1177/0038038519828906

[B87] Wu G. Chen Z. Liu J. Han D. Qiao B. (2021). Task assignment for social-oriented crowdsourcing. Front. Comput. Sci. 15,8. 10.1007/s11704-019-9119-8

[B88] Yates F. E. (2012). Self-Organizing Systems: The Emergence of Order. Denver, Colorado: Springer Science & Business Media.

[B89] Yin M. Suri S. Gray M. L. (2018). “Running out of time: the impact and value of flexibility in on-demand crowdwork,” in Proceedings of the 2018 CHI Conference on Human Factors in Computing Systems (Montreal), 1–11. 10.1145/3173574.3174004

[B90] Yin X. Wang H. Wang W. Zhu K. (2020). Task recommendation in crowdsourcing systems: a bibliometric analysis. Technol. Soc. 63:101337. 10.1016/j.techsoc.2020.101337

[B91] Yu D. Zhou Z. Wang Y. (2019). Crowdsourcing software task assignment method for collaborative development. IEEE Access 7, 35743–35754. 10.1109/ACCESS.2019.2905054

[B92] Zhou S. Valentine M. Bernstein M. S. (2018). “In search of the dream team: temporally constrained multi-armed bandits for identifying effective team structures,” in Proceedings of the 2018 Chi Conference on Human Factors in Computing Systems (Montreal), 1–13. 10.1145/3173574.3173682

